# Integrated genomics and proteomics analysis of *Paenibacillus peoriae* IBSD35 and insights into its antimicrobial characteristics

**DOI:** 10.1038/s41598-022-23613-y

**Published:** 2022-11-07

**Authors:** Ng Ngashangva, Pulok K. Mukherjee, Chandradev Sharma, Mohan C. Kalita, Indira Sarangthem

**Affiliations:** 1 A National Institute of Department of Biotechnology, Institute of Bioresources and Sustainable Development (IBSD), Govt. of India, Takyelpat, Imphal, Manipur 795001 India; 2 Department of Biotechnology, Gauhati University, Jalukbari, Guwahati, Assam 781014 India

**Keywords:** Biological techniques, Biotechnology, Computational biology and bioinformatics, Drug discovery, Microbiology, Molecular biology

## Abstract

Antimicrobial resistance has been developing fast and incurring a loss of human life, and there is a need for new antimicrobial agents. Naturally occurring antimicrobial peptides offer the characteristics to counter AMR because the resistance development is low or no resistance. Antimicrobial peptides from *Paenibacillus peoriae* IBSD35 cell-free supernatant were salted out and purified using chromatography and characterized with liquid chromatography–tandem-mass spectrometry. The extract has shown a high and broad spectrum of antimicrobial activity. Combining the strain IBSD35 genome sequence with its proteomic data enabled the prediction of biosynthetic gene clusters by connecting the peptide from LC–MS/MS data to the gene that encode. Antimicrobial peptide databases offered a platform for the effective search, prediction, and design of AMPs and expanded the studies on their isolation, structure elucidation, biological evaluation, and pathway engineering. The genome-based taxonomy and comparisons have shown that *P. peoriae* IBSD35 is closely related to *Paenibacillus peoriae* FSL J3-0120. *P. peoriae* IBSD35 harbored endophytic trait genes and nonribosomal peptide synthases biosynthetic gene clusters. The comparative genomics revealed evolutionary insights and facilitated the discovery of novel SMs using proteomics from the extract of *P. peoriae* IBSD35. It will increase the potential to find novel bio-molecules to counter AMR.

## Introduction

The discovery of penicillin has heralded the modern era of drugs and saved millions of lives. However, it was short-lived and antimicrobial resistance (AMR) has been developing fast and incurring a loss of human life^1,2^. Given this, there is a continuous need for new antimicrobial agents. Prokaryotes perform a vital function for organisms and ecosystems and provide by-products for human and veterinary medicine, agriculture, and manufacturing. They are well-known to mediate microbe-host and microbe-microbe interactions for their survival competition. Consequently, they produced diverse bio-molecules vital for the structure and proper function of living cells or organisms^3^. At the same time, they caused infectious diseases and extended the AMR through the environment, reproduction, and gene transfer^4^.

The first functionalized peptide-producing endophyte was isolated from *Kennedia nigriscans*^5^. Afterward, *Bacillus amyloliquefaciens* sp. was isolated from *Ophiopogon japonicas* which afforded the discovery of antitumor exo-polysaccharides^6^. We presumed that the plant-microbes association might have affected each other in their respective biochemical properties^7^. This interaction might enable microbe existence as an endophyte through absorbing or secreting biomolecules^8^. Furthermore, the discovery of anticancer taxol and taxane production by *Taxomyces andreanae* has stimulated research interest in bacterial and fungal endophytes^9^. The understudy *P. peoriae* IBSD35 (or strain IBSD35) was isolated from the stem of *Millettia pachycarpa* in our previous experiment^10–14^, and it has shown antimicrobial activity from its cell-free supernatant (CFS)^12^. Therefore, we investigated the source genome’s antimicrobial characteristics, extracellular antimicrobial compounds, and the potential to counter AMR.

Naturally occurring antimicrobial peptides (AMPs) offer the characters to counter AMR. They are present in every phylum and kingdom^15^ and are known for antibacterial, anticancer, antiviral, antifungal, antiparasite, effectors of innate immunity, and antibiotic adjuvant characteristics^16,17^. The resistance development of AMPs by gene mutation is less expected as mature peptides are created from a non-descript sequence of pro-peptides and lack a definite recognition site. They have multiple attacking target sites in contrast to common antibiotics that target specific proteins^16^. AMPs are highly conserved, hydrophobic, and mostly amphiphilic despite their diverse biological properties^18^.

A comprehensive genomics analysis, advanced proteomics techniques, AMP databases, and structural models offer a platform for effective search, prediction, drug development, and elaboration of AMPs^19,20^. The mass spectrometry (MS) technique can identify AMP sequence and mass from a mixture sample and subsequently disclose their primary, secondary, and tertiary structure^21,22^. The liquid chromatography-tandem-mass spectrometry (LC–MS/MS), proteomics, and AMP databases have broadly impacted AMP biology and the development of peptide-based drugs^23^. Moreover, genome mining has enabled the prediction of a biosynthetic gene cluster (BGC), linking the proteins and their peptides from LC–MS/MS data and connecting these natural products to the genes that encode them^24^. Proteomics has enabled genomic data to guide the discovery of new molecules to be applied in biomedical research^25–27^.

Therefore, the present study analyzes the characteristics of the *P. peoriae* IBSD35 draft genome and the proteomics of its extracellular AMPs. The understudy genome and its close strain comparisons illustrate its endophytic and antimicrobial traits. It provided an insight into *P. peoriae* IBSD35 molecular mechanisms and elucidated its potential as a source of novel AMPs to counter AMR.

## Results

### Genomic deoxyribonucleic acid (DNA) estimation and annotation

*Paenibacillus peoriae* IBSD35 inoculated on Luria Bertani (LB) agar plate was visible after 12 h of incubation at 38 °C. It is an endospore-forming and neutralophilic bacterium based on Integrated Microbial Genomes Annotation Pipeline (IMG AP) v.4.16.6^28,29^. The genomic deoxyribonucleic acid (gDNA) assessed with 0.8% agarose was estimated to be more than 50-kilo base pairs (kbp), and the concentration was 188.63 µg µl^−1^ (Supplementary Fig. S1). *P. peoriae* IBSD35 has a linear DNA topology with a total length of 5,862,582 bp. Its closest species reference genome is *Paenibacillus peoriae* KCTC 3763 with a symmetrical identity of 62.7495% and a symmetrical identity of 89.4884% with *P. peoriae *FSL J3-0120 in National Center for Biotechnology Information (NCBI) neighbor genome report^30^.

### Spectrum of antimicrobial activity of the *P. peoriae* IBSD35 inoculum

**Figure 1:**
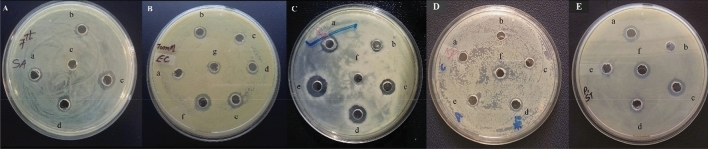
Cut-well agar diffusion showing antimicrobial activity against (**A**) *Staphylococcus aureus* ATCC 25923, (**B**) *Escherichia coli* ATCC 25922, (**C**) *Candida albicans* ATCC 10231, (**D**) *Klebsiella pneumoniae* ATCC 4352, and (**E**) *Salmonella typhimurium* ATCC 14028. a, b, c, d, e, and f indicated the cut-wells loaded at different steps of purification and concentration.

The CFS of *P. peoriae* IBSD35 inoculum retained antimicrobial activity against Gram-positive *Staphylococcus aureus* American Type Culture Collection (ATCC) 25923. The crude extract has shown antimicrobial activity against the Gram-negative *Escherichia coli* ATCC 25922 and fungal pathogen *Candida albicans* ATCC 10231, indicating it’s broad-spectrum of antimicrobial activity. The variation of antimicrobial activity at different stages of purification and concentration suggested the complex nature of biomolecules (Fig. 1A–C).

### Optimum growth conditions for antimicrobial metabolites production

**Figure 2:**
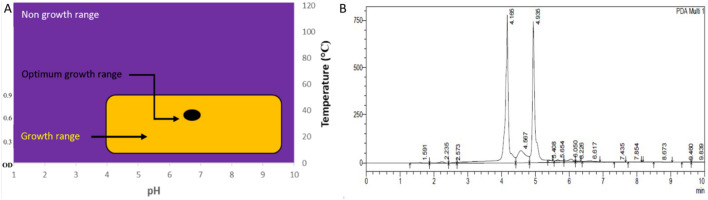
(**A**) The graphic diagram of the *P. peoriae* IBSD35 optimum growth curve in LB medium. The purple zone indicates the non-growth region, yellow zone represents the growth region, and the black dot indicates the optimum growth zone. (Left Y-axis is the optical density, Right Y- is the optimum temperature, and X-axis is the pH). (**B**) The chromatogram of the RP-HPLC. X-axis shows the retention time in min while Y-axis shows the absorbance. (The retention times indicate the peaks for practical purposes. 4.165 represents peak P4, and 4.567 represents peak P5). P4 retention time is 4.165 min. The P5 retention time is 4.567 min. Sharp peaks of P4 and P5 indicate they are sufficiently separated and have good biomolecule concentration. The P4 fractions exhibiting higher antimicrobial activity were pooled together and selected.

The *P. peoriae* IBSD35 inoculum’s optimum optical density (OD) was measured to be 0.65 at λ600 nm observed on the 6th day, coinciding with the exponential growth phase in the LB medium. The optimum growth was at pH 6.8–7. *P. peoriae* IBSD35 is a mesophile bacterium that grows best in moderate temperatures observed at 38 °C (Fig. 2A). The supernatant antimicrobial activity was determined as an arbitrary unit (AU) per milliliter which is the reciprocal of the highest dilution (2^n^) that resulted in the inhibition of *S. aureus* ATCC 25923 indicator lawns^31^. The AU was measured to be 1600 AU ml^−1^.

### Salting out and antimicrobial activity retention test of dialysate

The fraction (F4) eluted out with the 700 milimolar (mM) sodium chloride (NaCl) at the flow rate of 2 ml min^−1^ has shown antimicrobial activity against *S. aureus* ATCC 25923. The fraction OD was measured to be 1.147 and 2.803 at wavelength (λ)214 and λ280, respectively (Table S1). The fraction (F4) was collected, lyophilized into powder form, and store at − 20 °C. It dissolved in distilled water. It was dialyzed with 12.4 kilo dalton (kDa) molecular weight cut-off (MWCO) dialysis tube (Sigma Aldrich, Germany) to eliminate the larger protein complexes. The dialysate has retained antimicrobial activity against *S. aureus* ATCC 25923.

### Reverse phase-high performance liquid chromatography (RP-HPLC) and estimation of protein

The protein mixture gradient elution was at an isocratic flow rate of 2.5 ml min^−1^. The P4 (4.165) retention time was 4.165 min with a purity index of 0.86310, and the P5 (4.567) retention time was 4.567 min with a purity index of 0.95597 (Table S2). The RP-HPLC peaks P4 and P5 are sufficiently separated (Fig. 2B), and their absorption at λ205 and λ214 indicated the presence of amino acids and peptide bonds with other impurities. The absorbance at λ280 confirmed the presence of proteins. The P4 fractions had exhibited higher antimicrobial activity than P5. The purification fold of RP-HPLC was 151.72 starting from the initial crude extract (Table S1). The loss of bioactive metabolites from the harvesting of the cells ranged from 0 to 7.7%. The loss of cells after extraction ranged from 0.6 to 2.3%.

### Effect of temperature, detergents, inorganic solvents, pH variation, and degradative enzymes on antimicrobial activity

A 0.2 µm filter (Avixa) sterilized supernatant treated at different temperatures retained the antimicrobial activity against *S. aureus* ATCC 25923. Autoclaving at 121 °C for 15 min and treatment with Tween 20, Tween 80, and sodium dodecyl sulfate (SDS) have slightly increased the antimicrobial activity (from 10 to 14 mm zone of inhibition diameter). It might be due to the disaggregation and exposure of peptide bonds. The inorganic solvents, pH variation, and degradative enzymes treated at a 1:1 ratio with the filtrate did not affect the antimicrobial activity significantly. The antimicrobial activity of the CFS is almost comparable to the ampicillin at 1 mg ml^−1^ (Table S3). Moreover, the sample persisted in low-temperature freezer storage (− 20 °C) without any preservative for 1 year.

### Mass spectrometry and proteomics analysis of peptide mixtures

The *P. peoriae* IBSD35 proteins searched with Sequest, Mascot, and PEAKS from the uninterpreted experimental LC–MS/MS database confirmed the prediction of AMPs (Supplementary LC–MS Experiment data)^32^. The number of sequence protein entries predicted in the *P. peoriae* IBSD35 Uniprot database is 15 protein groups. Different peptide sequences have detected during the process (Table 1). An uncharacterized protein was predicted from the experimental LC–MS/MS with accession no. A0A2S6NUT6. The Uniprot search has shown that it is 94.87% similar to A0A509FZN2_PAEPO of *Paenibacillus polymyxa* (A0A509FZN2) (Bit score-296, E-value-3.6 e−89)^33^. Combining the genome mining and proteomics analysis has shown that the gene (ID 2816869382) is 97% similar to the gene (ID 2556935736) of *Paenibacillus polymyxa* CR1 (NC_023037) (Bit score-286; 1e−95) and it has 134 amino acid residues.

**Table 1:** The peptide sequences, masses, m/z, PTM, and their similarity percentage with the known peptide.

Peptide	m/z	Mass	PTM	AMP	Similarity (%)
ACATEYTAK	479.3219	956.4273	N	Fusaricidin D	40
MFPPVVVYK	540.3121	1078.5884	N	Bacteriocin N5-2	35.71
MTTNYTSK	473.3067	944.4274	N	Fusaricidin D	37.5
AC (+ 57.02) TVDVHK	465.307	928.4437	Y	Lipopeptide	37.5
AGTLPEPLAQDGLVYR	850.4984	1698.894	N	Lassomycin	41.17
ATAGGVGLM(+ 15.99)SAQTAEGAMNEVCAMLTR	886.0884	2655.2124	Y	Macedovicin, lantibiotic	36.66
KCTLEVHK	479.3219	956.5113	N	Colistin (Polymyxin E1 and E2	33.33
K(+ 42.01)ANPLSTGK	479.3219	956.5291	Y	Duramycin C (Lantibiotic, type B, class 1 bacteriocin	36.84
MESEDHIScLPYTNHVSRSTTVTSLNSHTYTLTFPTEISQR	982.4637	4685.13	Y	Ericin	32.6
AAGIQAQAGFGLSDSIQGTGKQKCSFCK	2858.40070	2802.17	C	Fermentin	38.23

The AMP sequences of the *P. peoriae* IBSD35 which have putative BGCs in the genome were generated using LC–MS/MS.

*m/z* mass to a charge ratio, *PTM* post translational modification, *AMP* antimicrobial peptide, *%* percentage.

**Figure 3:**
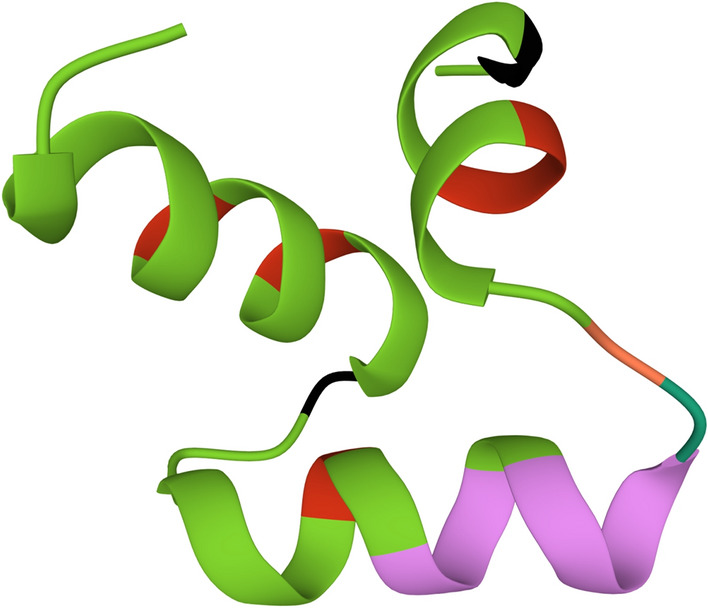
A 3-D model of peoriaerin IBSD35 predicted using de novo modeling. The amino acids composition of peptide is AAGIQAQAGFGLSDSIQGTGKQKCSFCK. Sequence contains high Gly content. It is indicated in black color. Glycine is integral to the formation of alpha-helices in secondary protein structure due to its compact form. The purple color indicated the helix structure property. It included the green color which intersects with the purple color. The green color indicated the residues embedded in membrane. The light orange color indicated the Trp which is the rings in the residues. The red color indicated the Ala. The whole structure represented the backbone and side chain of the AMP except the Gly indicated in black color.

An AMP predicted from the protein lists with accession no. A0A2S6NUT6 is designated peoriaerin IBSD35 based on its source name (Table 2). Its sequence is AAGIQAQAGFGLSDSIQGTGKQKCSFCK. The N-terminal of the sequence considered is alanine (Ala). It has 28 amino acid residues with a molecular weight (MW) of 2802.17 Da (Supplementary Fig. S2). Its sequence backbone has a large portion of helix and residues embedded in the membrane. It suggested peoriaerin IBSD3 might insert the membrane for its antimicrobial activity (Fig. 3). The peptide sequence alignment indicated that it is 38.23% similar to fermentin^20^. The three-dimensional (3-D) model of peoriaerin IBSD35 was generated using an automated protein structure de novo modeling and deposited at ModelArchive with accession no. 10.5452ma-id7l4 (Fig. 3).

**Table 2:** Protein group accession list from LC–MS data and the peptide sequence generated from MS-ion search.

Accession no.	Description	Peptide	Modification	Charge	MH + [Da]	ΔM [ppm]	RT [min]	# Missed cleavages
A0A2S6NUT6	Uncharacterized protein OS = Paenibacillus peoriae OX = 59893 GN = C5G87_15860 PE = 4 SV = 1 − [A0A2S6NUT6_9BACL]	AAGIQAQAGFGLSDSIQGTGKQKCSFcK	C27(Carbamidomethyl)	5	2858.40070	2.86	39.06	2

### Peoraerin IBSD35 mode of action imaging through scanning electron microscopy

**Figure 4:**
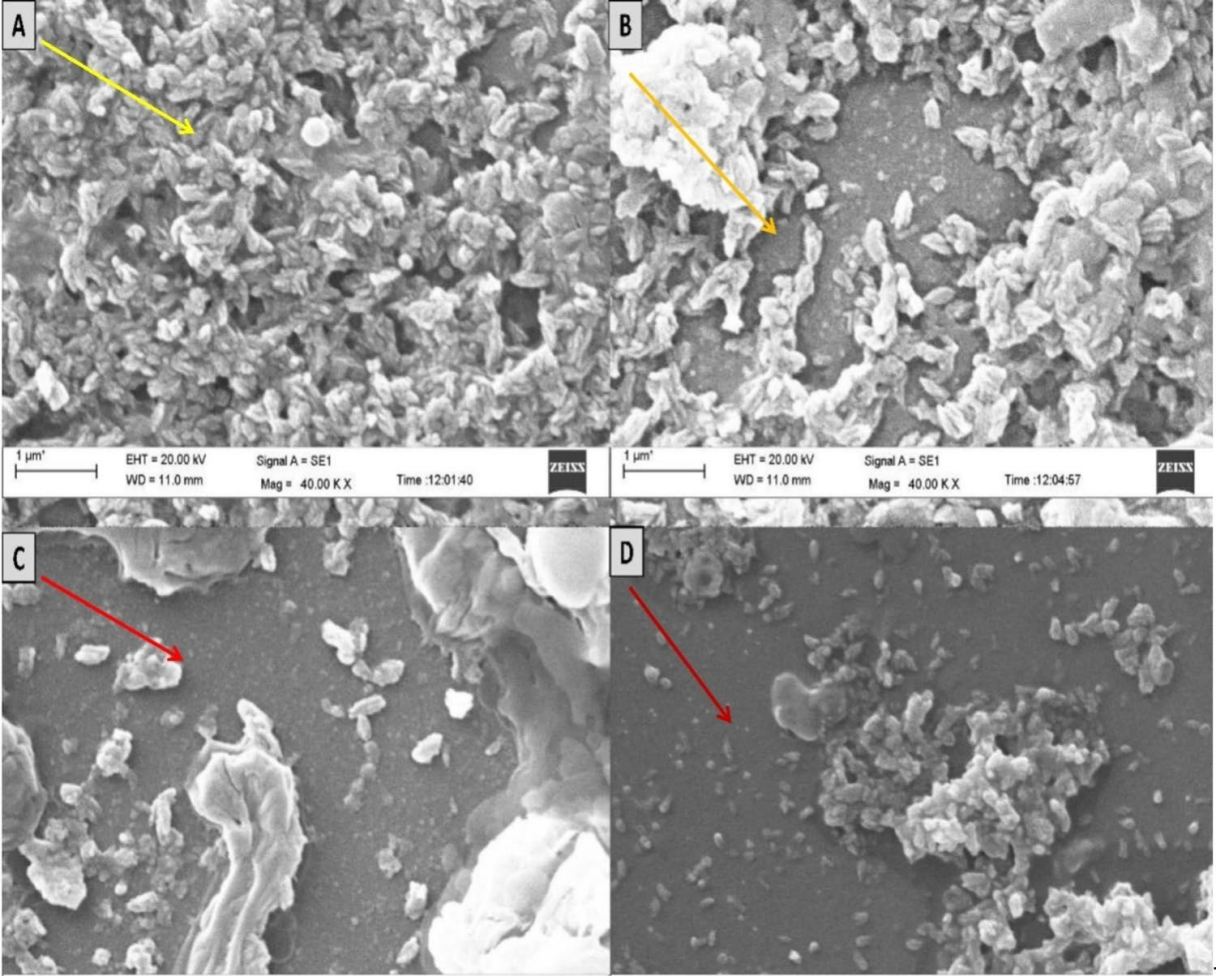
Mode of action of peoriaerin IBSD35 against *S. aureus* ATCC 25923 and imaging with scanning electron microscopy. The morphological changes of *S. aureus* ATCC 25923 when treated with peoriaerin IBSD35 1× MIC at different experimental conditions. (**A**) *S. aureus* ATCC 25923 only, (**B**) *S. aureus* ATCC 25923 with peoriaerin IBSD35 at 0 h, (**C**) *S. aureus* ATCC 25923 with peoriaerin IBSD35 at 1 h, (**D**) *S. aureus* ATCC 25923 with peoriaerin IBSD35 at 2 h. The yellow arrows point to *S. aureus* ATCC 25923 colonies without peoriaerin IBSD35 and at zero hours with peoriaerin IBSD35. The red arrow points to the disappearance of *S. aureus* ATCC 25923 at 1 and 2 h.

The antimicrobial action had observed after 1 h of incubation. The minimum inhibition concentration (MIC) of peoriaerin IBSD35 was determined to be 0.04 µg µl^−1^. Scanning electron microscopy (SEM) imaging indicated that the pathogen colonies were slowly disappearing. It correlated with the decreased colony count from more than 400 to 4 in 2 h (Fig. 4). The OD at λ600 nm was 1.409 indicating the cell growth and debris turbidity. There was no evidence of DNA and protein leakage. It showed that the AMP might kill *S. aureus* ATCC 25923 by penetrating the cell cytoplasm and periplasm.

### Draft genome sequencing, assembly and annotations of *P. peoriae* IBSD35

**Figure 5:**
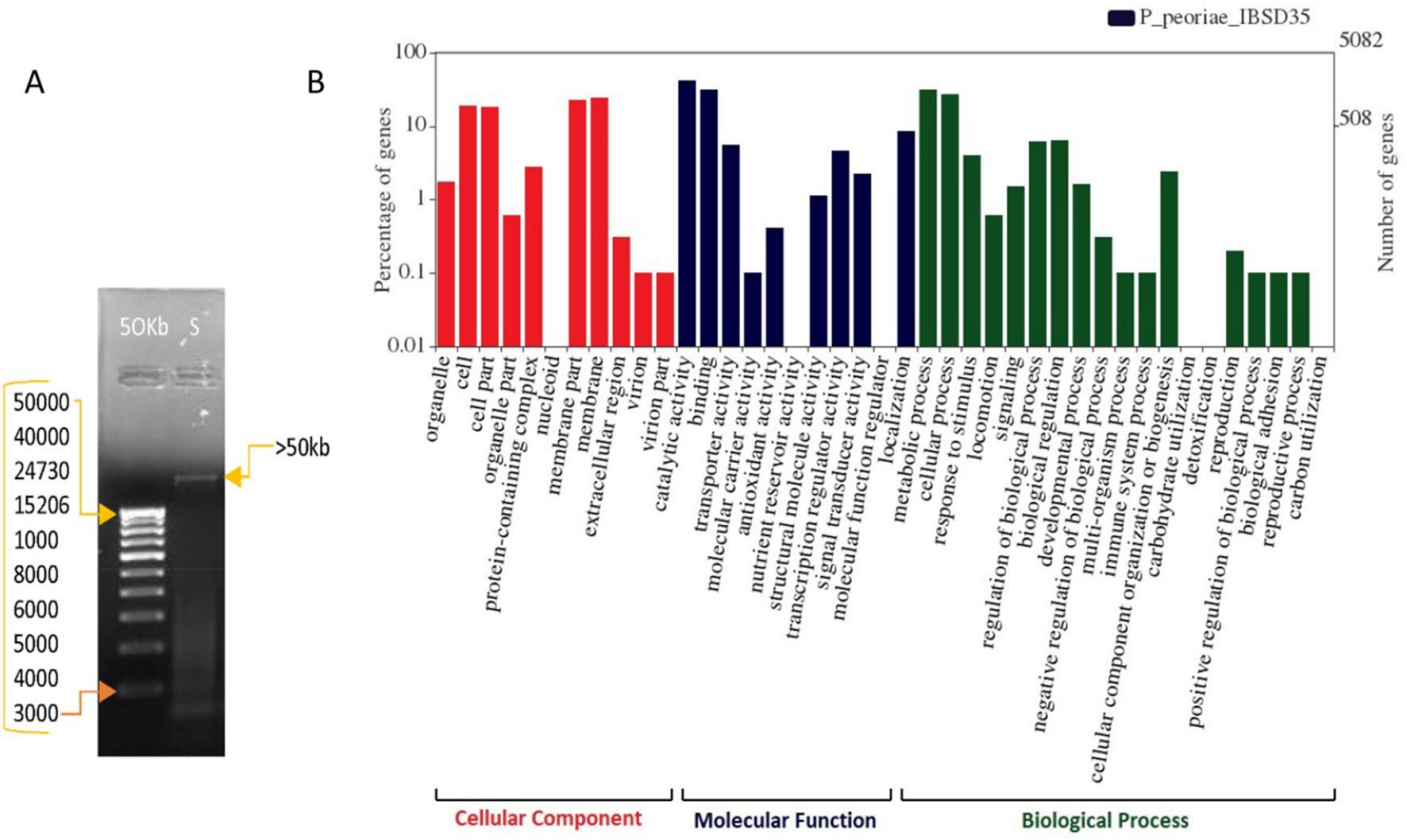
(**A**) The crude gDNA band quality checked on 0.8% agarose gel, where Marker lane is 50 kb DNA ladder and, lane S is DNA whose molecular weight is more than 50 kb. (**B**) The WEGO analysis of *P. peoriae* IBSD35 genome. The gene ontology of genome has been classified into cellular component (Red), molecular function (Blue), and biological process (green) categories. The numbers of the genes are indicated in percentage.

The nucleic acid and protein ratio (A260/A280) was estimated to be 1.9 (Fig. 5A). The draft genome with 250 bp-end reads produced a total number of raw reads (R1 + R2) of 15,497,242 bp^34^. Total bp are 3,874,320,000 bp with 46.01 guanidine-cytosine (GC) % and a read length of 250. The clean reads produced a total length of 5,862,582 bp with 58.0× fold coverage. The *de-novo* whole genome assembled with ABySS v.2.0^35^ has produced 66 contigs totaling 5,862,582 bp with N50 of 400,732 and L50 of 6, 42 scaffolds and a GC content of 45.6% (Table S4). The NCBI Prokaryotic Genome Annotation Pipeline (PGAP) v.4.4 has identified 5245 genes, 4983 coding sequences (CDSs), 9, 2, 3 (5S, 16S, 23S) rRNAs (ribosomal ribonucleic acids), 2, 1 (5S, 16S) complete rRNAs, 7, 1, 3 (5S, 16S, 23S) partial rRNAs, 81 tRNAs (transfer ribonucleic acids), and 4 nc RNAs (noncoding RNAs) (Table S4). CRISPR/csd (clustered regularly interspaced short palindromic repeats) is involved in the prokaryotic defense system^36^. Out of two 16S rRNA bacterial short sequence units (SSU), one has 1560 bp, and the other has 45 bp based on IMG AP v.4.16.6.

### Global overview of *P. peoriae *IBSD35 genome

The maximum number of protein-coding genes (4983) is related to the genetic information process (47%), environment information processing (45%), carbohydrate metabolism protein family (42%), and signaling process (31%) (Supplementary Fig. S3)^37,38^. Membrane and cell contributed the highest percentage of genes in the cellular component category, while the catalytic activity, binding, and transportation account for the highest number in molecular function. The metabolic and cellular processes have the highest percentage within the biological processes category (Fig. 5B). *P. peoriae* IBSD35 harbored cytochrome bd ubiquinol oxidase [EC: 7.1.1.7], a component of the aerobic respiratory chain gene (*cyd*A). The ModelSEED predicted its reaction in the cytosol as^39^

1 $${\text{Ubiquinol }}\left( {{8 } + { 2}.{\text{5 H }} + \, 0.{\text{5 O}}_{{2}} \Leftrightarrow {\text{ Ubiquinol-8 }} + {\text{ H}}_{{2}} {\text{O }} + { 2}.{\text{5 H}}} \right).$$

*Paenibacillus peoriae* IBSD35 genome harbored nitrogenase molybdenum-iron protein beta chain (*nif*K, *nif*D) [EC: 1.18.6.1]^40,41^, phosphotransferase system (PTS), and sugar-specific component. They might be utilizing extra-cellular trehalose, cellobiose, and maltose. It revealed the *P. Peoriae* IBSD35 auxotrophic lifestyle (www.kegg.jp/kegg/kegg1.html)^42,43^. The Joint Genome Institute-Integrated Microbial Genomes and Microbiomes System (JGI-IMG/M) analysis revealed a putative horizontally transferred genes count of 10 (0.19%) with the best hits to genes from *Proteobacteria* and *Clostridia*. The trans-membrane count is 1392 (26.28%) and might serve as a gateway to transport specific substances across the membrane^28,29^. The signal peptide is 303 (5.72%) of the total CDS identified. It might prompt the translocation of a cell protein secretion^44,45^.

*Paenibacillus peoriae* IBSD35 harbored *Nis*K-*Nis*R biosynthesis sensor histidine kinase [EC: 2.7.13.3], a two-component signal transduction system (TCS), known to affect the biosynthesis of AMP^46,47^. It also harbored sensor histidine kinase and response regulators, two *Des*K–*Des*R of the NarL family, four *Che*A–*Che*Y of a chemo-taxis family, two *Res*-*Res*D, and two *Deg*S-*Deg*U for motility, regulation, and interactions. Flagellar proteins (fliS) in the cellular chaperones might cause microbe motility and signal transduction. A flagellin/flagellar hook-associated protein (*fli*C) includes glycerol kinase and molecular chaperone (HtpG) that might involve in plant–microbe interaction^48^. *P. peoriae* IBSD35 genome also harbored biodegradation capability proteins for xenobiotic compounds^49^.

### Comparison of genomes characteristics

The selected *Paenibacillus* species have shown varying numbers of DNA scaffolds ranging from 1 to 56. *Paenibacillus* sp. JDR-2 has the largest genome size (7,184,930 bp), and *B. subtilis subtilis* AG1839 has the smallest genome size (4,193,640 bp) among the selected genomes (Table 3). The dot plot homology revealed a low level of *P. peoriae* IBSD35 synteny with *Paenibacillus peoriae* KCTC 3763 but a higher synteny with *Paenibacillus peoriae* HS311, *P. polymyxa* SC2, and *P. polymyxa* E681 (Supplementary Fig. S4). The predicted numbers of nonribosomal peptide synthase (NRPS) BGCs ranged from 0 to 18 among the eleven genomes indicating their diverse and rich SM natural products (Table 3). *P. peoriae* IBSD35 harbored 18 NRPS (72% of the total 25 BGCs) and encompassed the highest number of NRPS BGCs among the selected genomes comparisons.

**Table 3:** Comparative genome features.

Gene count	Scaffold count	GC count	CDS count	16S rRNA count	COG count	Signal peptide count	Signal peptide %	Transmembrane count	Transmembrane %	Horizontally transferred count	Biosynthetic cluster count	Genome size	Genome
5234	49	2,680,707	5113	6	3330	403	7.7	1379	26.35	121	17	5,771,987	1
4932	1	2,470,325	4805	12	3076	372	7.54	1273	25.81	173	13	5,394,884	2
5296	42	2,666,126	5187	2	3372	303	5.72	1392	26.28	10	25	5,852,622	3
6228	2	2,785,063	6032	14	3314	397	6.37	1506	24.18	257	12	6,241,931	4
5577	2	2,829,598	5425	13	3494	299	5.36	1435	25.73	9	14	6,219,815	5
5993	56	3,116,495	5926	3	3509	435	7.26	1701	28.38	177	9	6,385,945	6
4347	1	1,823,585	4231	10	2934	280	6.44	1163	26.75	1	12	4,193,640	7
8325	3	6,519,342	8210	6	4575	750	9.01	1896	22.77	98	31	9,054,847	8
5642	1	2,845,207	5525	9	3501	410	7.27	1422	25.2	47	12	6,083,395	9
6410	1	3,612,449	6288	12	4096	620	9.67	1889	29.47	708	4	7,184,930	10
5407	1	2,746,181	5283	13	3392	298	5.51	1383	25.58	68	13	6,024,666	11

1. *P. peoriae* KCTC 3763 (NZ_AGFX01000000) 2. *P. polymyxa* E681 (NZ_CP048793) 3. *P. peoriae* IBSD35 (*NZ_PTJM01000000*) 4. *P. polymyxa* SC2 (NC_014622) 5. *P. peoriae* HS311 (NZ_CP011512) 6. *P. vortex* V453 (*NZ_ADHJ01000000*) 7. *B. subtilis subtilis* AG1839 (NZ_CP008698) 8. *S. coelicolor* A3(2) (AL645882) 9. *P. terrae* HPL-003 (NC_016641) 10. *Paenibacillus* sp. JDR-2 (NC_012914), and 11. *P. polymyxa* CR1 (NC_023037).

### Antibiotics and other secondary metabolites (SMs) gene cluster of *P. peoriae* IBSD35

The CFS of *P. peoriae* IBSD35 has shown a high and broad-spectrum antimicrobial activity against *S. aureus* ATCC 25923, *E. coli* ATCC 25922, *C. albicans* ATCC 10231, *Klebsiella pneumoniae* ATCC 4352, and *Salmonella typhimurium* ATCC 14028 (Fig. 1A–E). The activity was comparable with ampicillin and nisin at a much lower dose. Antibiotics and Secondary Metabolite Analysis Shell (AntiSMASH) v5 using Integrated Microbial Genomes Atlas of Biosynthetic gene clusters (IMG-ABC) web server tools predicted *P. peoriae* IBSD35 harbored 25 BGCs^50,51^. LC–MS sequencing of RP-HPLC purified sample have predicted 15 protein lists (Table 3).

*Paenibacillus peoriae* IBSD35 harbored resistance genes for beta-lactam, vancomycin, cationic antimicrobial peptide (CAMP), platinum drug, and antifolate drug^52–55^. The Rapid Annotation of microbial genomes using Subsystems Technology (RAST) tool predicted that *the P. peoriae* IBSD35 genome harbored 29 compounds resistant to antibiotics and toxic compounds as compared to the metabolic reconstruction of *P. polymyxa* E681. They shared 23 compounds, while one is unique in the *P. peoriae* IBSD35 and nine are unique in *P. polymyxa* E681.

**Figure 6:**
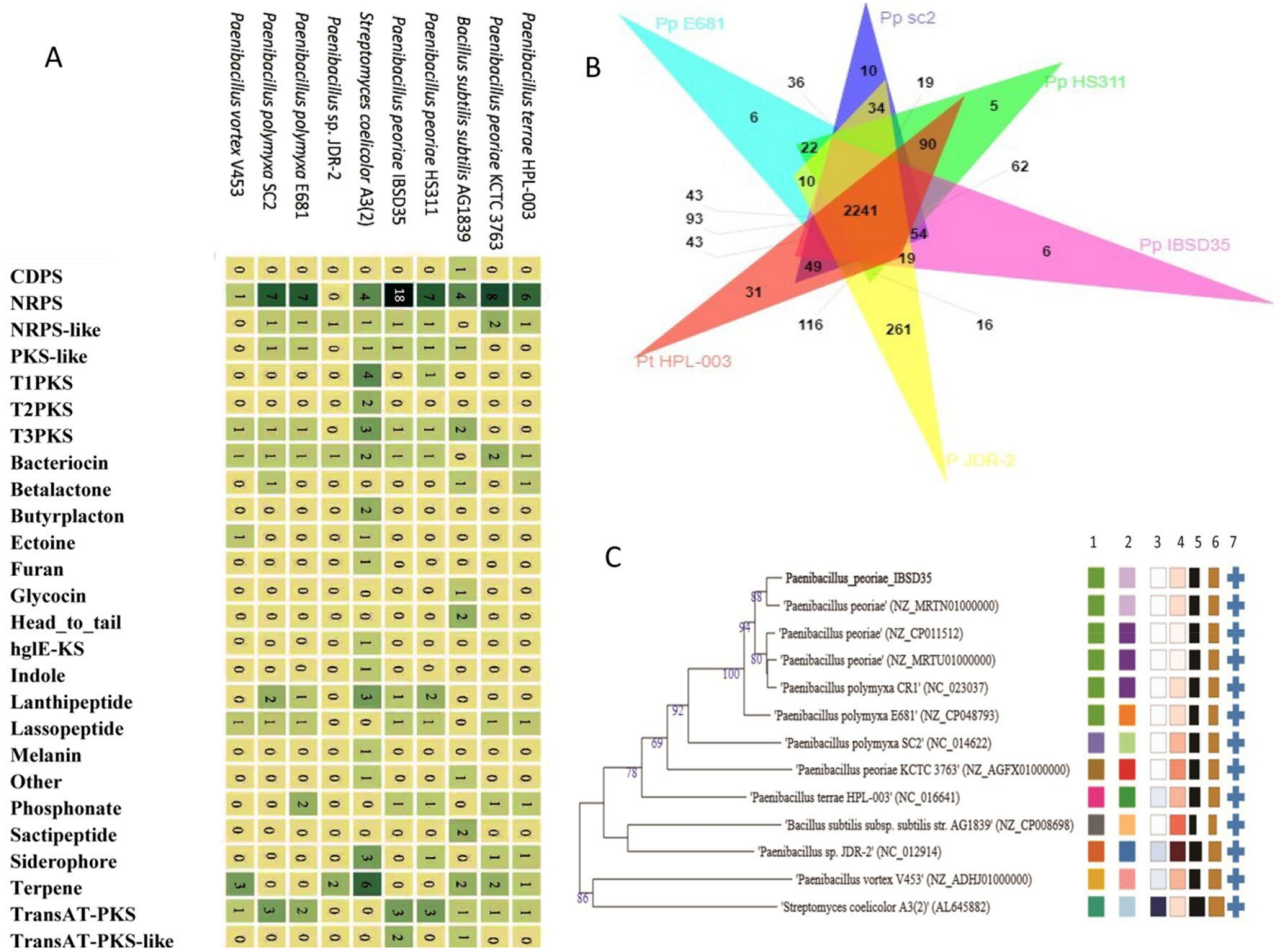
(**A**) The Heatmap of the BGCs of the selected genome. The names of the genomes are indicated on the left side. The rows represent the genomes BGCs, and its columns indicate the type of BGC. The BC types are indicated in description on bottom. Cells are colored with hues of green based on the number of copies of the selected protein family (Pfam) in the BC (numbers of BCs are indicated in numbering). Pfams (columns) that occur in all BCs (rows) define the core functions. (**B**) Venn diagram for the comparison of six closely related strains. (*P. terrae* HPL-003(NC_016641), *P. peoriae* HS311 (NZ_CP011512), *P. peoriae* IBSD35 (*NZ_PTJM01000000*), *Paenibacillus* sp. JDR-2 (NC_012914), *P. polymyxa* E681(NZ_CP048793), and *P. polymyxa* SC2 (NC_014622). They formed 5615 clusters, 5296 orthologous clusters, and 2125 single-copy gene clusters. They shared 2241 gene clusters. 6-clusters are specific to *P. peoriae* IBSD35. (**C**) Tree inferred with FastME 2.1.6.1^114^ from GBDP distances calculated from genome sequences. *P. peoriae* IBSD35 has a dDDH of (d4) 76.1, (C.I.) d4 of [73.1–78.9], and a difference G + C% of 0.16 with *P. peoriae* HS311 indicating their close species relation. The branch lengths are scaled in terms of GBDP distance formula d5. (Leaf labels are annotated by affiliation to species (1) and subspecies (2) clusters, genomic G + C content (3), δ values (4), overall genome sequence length (5), number of proteins (6), and the kind of strain (7). User-provided GenBank accession IDs are shown in parentheses; master record accessions are truncated (Percent G + C—43.5–72.1, No of Proteins—4218–7738, Genome size—4,193,640–8,667,507 bp), delta statistics—0.22–0.373, SSU lengths (in bp)—1331–1546).

*Paenibacillus peoriae* IBSD35 harboring 18 NRPS indicated that nonribosomal peptide compounds are the most abundant SMs of our understudy strain IBSD35 genome (Fig. 6A). The genome encompassed three gene clusters- one Trans-acyltransferase polyketide synthase (Trans AT-PKS) and two clusters of Trans AT-PKS. Two BGCs are associated with Butyrolactone, and one each for Type III-polyketide synthase (T3PKS), bacteriocin, phosphonate, lanthipeptide, NRPS-like, polyketide synthases-like, and lasso peptide. At least 12 BGCs start from a position, and 2 BGCs code rhizomides A, B, and C as predicted by the Antibiotics and Secondary Metabolite Analysis Shell-Minimum Information about a Biosynthetic Gene Cluster (AntiSMASH-MIBiG) comparison (Table 4).

**Table 4:** JGI-ABC using AntiSMASH v 5.0 predicted the putative BGCs coding for antibiotics and secondary metabolites.

AntiSMASH Cluster	Type	From bp	To bp	Most similar known gene cluster	Similarity
1	Nrps	44,661	96,334	Fusaricidin biosynthetic	100%
2	Nrps	1	1886	–	
3	Transatpks-Nrps	1	43,034	–	
4	Nrps	1	19,240	–	
5	Lantipeptide	196,869	223,875	Mersacidin biosynthetic	20
6	Nrps	1	7725	–	
7	Nrps	1	2793	–	
8	Nrps	1	8875	–	
9	Nrps	1	47,371	Tridecaptin biosynthetic	40
10	Nrps	1	23,231	Tridecaptin biosynthetic	80
11	Other	348,320	382,015	–	
12	Nrps	50,170	83,876	–	
13	Nrps	1	5339	–	
14	Nrps	1	2640	–	
15	Nrps	1	25,403	–	
16	Nrps	1	4621	–	
17	Nrps	1	34,116	–	
18	Lassopeptide	91,874	115,990	–	
19	Transatpks-Nrps	151,159	251,144	–	
20	Bacteriocin	291,553	301,789	–	
21	Transatpks	1	55,964	Bacillaene biosynthetic	28
22	Transatpks	1	8497	Misakinolide biosynthetic	12
23	Transatpks-Others-Nrps	1	34,957	Nosperin biosynthetic	46
24	Nrps	1	14,018	Polymyxin biosynthetic	100
25	Phosphonate	165,862	206,761	–	

The clusters include NRPS cluster such as transatpks, bacillaene, misakinolide, and nosperin biosynthetic; other unspecified ribosomally synthesized and post-translationally modified peptide product (RiPP) cluster; Transatpks-NRPS, and Lassopeptide cluster. (AntiSMASH cluster numbers, region, similarity % and similar known cluster are shown, – indicates which do not have similar cluster in the database).

### Orthologous gene clusters of *P. peoriae* IBSD35 genome

OrthoVenn analysis has shown the selected *Paenibacillus* species (*P. peoriae* HS311, *Paenibacillus* sp. JDR-2, *P. polymyxa* E681, *P. polymyxa* SC2, *P. terrae* HPL-003, and *P. peoriae* IBSD35 formed 5615 clusters, 5296 orthologous clusters (at least contains two species), and 2125 single-copy gene clusters (E-value = 1e−2 and inflation value = 1.5) (Fig. 6B). The OrthoVenn diagram has shown that all 6 *Paenibacillus* species have shared 2241 gene clusters suggesting their conservation in the lineage. Additionally, it has 6-clusters specific to *P. peoriae* IBSD35 that might be lineage-specific gene clusters. The single-copy genes have suggested they maintained single-copy status through evolutionary time after the divergence of these species.

### Central carbon metabolism (CCM) of *P. peoriae* IBSD35 genome

The metabolite precursors of *P. peoriae* IBSD35 cell biomass included gluconeogenesis (GG), citrate cycle (TCA), pentose phosphate pathway (PPP) metabolites, and pyruvate metabolism (Table 5)^43,56–58^. The precursors of polyketide-malonyl Coenzyme A (CoA) generated from the pyruvate metabolism- the Acetyl CoA included propinol CoA, and butanol CoA have derived from fatty acid biosynthesis and amino acids metabolisms^59^. The strain IBSD35 might generate cell energy-adenosine triphosphate (ATP) and reducing agents. The reaction equation of ATP in the cytosol of *P. peoriae* IBSD35 predicted from the ModelSEED is^37,39^

2 $${\text{ATP }}\left[ {{\text{c}}0} \right] \, + {\text{ NAD }}\left[ {{\text{c}}0} \right] \, < = > {\text{ NADP }}\left[ {{\text{c}}0} \right] \, + {\text{ ADP }}\left[ {{\text{c}}0} \right] \, + {\text{ H}} + \, \left[ {{\text{c}}0} \right] \, \left( {{\text{c}}0} \right).$$

**Table 5:** The main precursor metabolites identified from the *P. peoriae* IBSD35 genome using KEGG pathway database analysis.

Metabolites	Building block produced	Pathway
2-Oxoglutarate	Arginine	Citrate cycle
Oxaloacetate	Alanine, aspartate, glutamate	Citrate cycle
Acetyl-coA	Fatty acid and ketone bodies, tryptophan, phenylalanine, and tyrosine	Citrate cycle
Homocitrate (Acetyl-coA)	Lysine	(Pyruvate metabolism) citrate cycle/glycolysis
3-Carboxy-3-hydroxy-4methylpentanoate	Leucine	(Pyruvate metabolism) citrate cycle
S-2-acetolactate (pyruvate)	Valine, leucine and isoleucine	Glycolysis
Fumarate	Arginine	Citrate cycle
D-ribose 5P	Nucleotides and histidine metabolism	Pentose phosphate pathway

### Genome-scale phylogeny analysis

*Paenibacillus peoriae* IBSD35 had a digital DNA-DNA hybridization (dDDH) of Genome-To-Genome Distance Calculator (GGDC) formula 2 (d4) 76.1, confidence intervals (C.I.) d4 of [73.1–78.9], and a difference G + C% of 0.16 with *P. peoriae* HS311 indicating their close species relation. *P. peoriae* IBSD35 has a dDDH of d4 86.2, C.I. d4 of (83.6–88.5), and a difference G + C% of 0.00 with *P. peoriae* FSL J3-0120 which indicated their very close relationship. Additionally, *P. polymyxa* CR1 and *P. peoriae* IBSD35 exhibited a close relationship with d4 75.6, C.I. [72.6–78.4] and a difference G + C% of 0.02 (Fig. 6C). The strain IBSD35 exhibited OrthoANI values of 96.37 with *P. peoriae* FSL H8-0551which is above the species boundary value of average nucleotide identity (ANI) = 95–96%. The result indicated that the G + C% difference computed from the query and subject genome sequences varied by no more than 1% and proved they are within species.

### Commensal lifestyle of *P. peoriae* IBSD35

The genome was predicted to harbor sugar-specific component phenotypes, thus possibly utilizing the host plant trehalose, l-arabinose, sucrose, and maltose. It harbored Tat A (twin-arginine translocation) and Sec (general secretory pathway) protein secretion systems for transportation across the cytoplasmic membrane^60,61^. Most secretion systems genes related to virulence and pathogenicity, such as types I, II, III, V, and VI secretion systems^62^, were absent in the *P. peoriae* IBSD35 genome, which supported its non-pathogenicity characters while handling.

### Nitrogen fixation characters of *P. peoriae* IBSD35

It harbored putative genes encoding Mo-nitrogenase MoFe protein (EC 1.18.6.1), multi-functional system (MFS) transporter, nitrate/nitrite transporter, and ammonium transporter involved in the uptake of nitrate and nitrite (Supplementary Figs. S5, S6). The nitrogen fixation genes include *nar*G, *nar*H, *nar*I, *nar*J, *nar*W, *nir*A, *nir*B, *nas*A, *hcp*, *gdh*A, *isc*U, *nif*B, *nif*D, *nif*E, *nif*K, *nif*N, *nif*E, *nif*H, *nif*V, *nif*X, *nif*Z) and other putative nitrogen-fixing proteins plus a hypothetical protein that lacks an assigned function were identified using the Kyoto Encyclopedia of Genes and Genomes (KEGG) pathway database analysis which indicated its nitrogen-fixation nature (Table S5) (www.kegg.jp/kegg/kegg1.html). However, the nitrogen fixation regulator (*nif*L/A) was not visible in the nitrogen metabolism pathway^63^. *P. peoriae* IBSD35 might absorb some of the nitrate sources from the host plant.

### Plant-associated endophyte characters of *P. peoriae* IBSD35

The proteins involved in the flagellum assembly gene ontology (GO): 0044780, flagellum-dependent cell motility (GO: 0071973), and chemotaxis (GO: 0006935) identified from in silico genome analysis of *P. peoriae* IBSD35 confirmed the bacterium motility traits. It harbored TCS CheA/CheY, methyl-accepting chemoreceptor proteins (mcp, K03406), an adaptor protein CheW (K03408), and flagellar motor switch protein FliM (K02416). They are the core of bacterial chemotaxis signaling genes. They are known to involve in colonization and interaction with host plants^29,46,48,49^. The analysis also revealed cellular motility proteins for the resources transport and substrate-binding protein of ribose (*rbs*B, K10439). These traits are required for mobility and thriving in heterogeneous and highly competitive environments. Endoglucanase [EC: 3.2.1.4] enzyme was visible in the understudy genome, and it is necessary for spreading to other intracellular tissues to enter the endophytic life stage (www.kegg.jp/kegg/kegg1.html).

## Discussion

Antibiotics have revolutionized the treatment of tropical infections, communicable diseases, food-borne, and other poverty-related infectious diseases. However, the efficacy is short-lived and turned into a resilient clinical problem eliciting innumerable public health crises and causing economic burden^1,2,4^. Therefore, there is a continuous need for a new antimicrobial agent with high specificity, efficacy, efficiency, and anti-resistant to tackle the AMR challenge^2,64^. AMPs have prospects because of their antibacterial, anticancer, antiviral, antifungal, anti-aging, and anti-parasite, regulating cell proliferation, extracellular matrix production, cellular immune responses, and adjuvant characteristics^15,16,65^. Lately, many important SMs have reported from endophytes of medicinal plants from less explored sources which offered unprecedented opportunities to find novel antimicrobial agents^5,6^. Moreover, the availability of ever-increasing genome sequences, a comprehensive proteomics database, and advanced bioassay techniques offer a platform for the dereplication of old compounds, effective search, prediction, and design of novel AMP to counter AMR^19,20^.

*Paenibacillus peoriae* IBSD35 from the stock solution has retained antimicrobial activity against *S. aureus* ATCC 25923, indicating its inherent antimicrobial potential^66^. Endospore forming traits may help its endophytic association with the host plant^67^. Its neutralophilic characters might help neutralize its cytoplasm relative to the plant cell to meet the pH challenges. It might be through direct active uptake or efflux of protons from the cells or tissues^29^. These indirectly suggested host-plant physiology as well. The optimum growth condition of the strain IBSD35 recorded at 38°, and pH 6.8–7 in LB medium indicated the mesophilic traits. The cultivable characters confirmed *P. peoriae* IBSD35’s ability to live as a free-living bacterium in laboratory conditions.

An antimicrobial protein mixture precipitated from LB fermentation broth with 70% ammonium sulfate suggests that at least one or more of the putative 25 BGCs of *P. peoriae* IBSD35 might express antimicrobial metabolites. The 700 mM NaCl eluent from DEAE-C column chromatography has retained activity against *S. aureus* ATCC 25923. The RP-HPLC peaks P4 and P5, absorption at λ205 and λ214, suggested the presence of amino acids and peptide bonds with other impurities. The absorbance at λ280 confirmed the presence of proteins^68,69^. Antimicrobial protein total yield was low at 0.05%, which can be enhanced using an advanced purification system or by up-regulating its BGC expression. MIC of peoriaerin IBSD35 at 0.04 µg µl^−1^ indicating its high specific activity and potency against *S. aureus* ATCC 25923 compared with ampicillin and nisin at 1 mg ml^−1^.

The purified AMP has shown antimicrobial activity against *S. aureus* ATCC 25923, *E. coli* ATCC 25922, *C. albicans* ATCC 10231, *K. pneumoniae* ATCC 4352, and *S. typhimurium* ATCC 14028 connoted its broad-spectrum antimicrobial character (Tables S6, S7). The sample has retained activity after prolonged storage, and its resistance to degradative enzymes indicated its potential for drug development against AMR. Antimicrobial activity was altering at different stages of purification. It might be due to the disintegration of proteins. The treatment of SDS and high-temperature slightly increased the activity. It might be from the disintegration or aggregation of the proteins and other biomolecule complexes (Table S3). The sample can withstand autoclaving at 121 °C for 15 min presenting a potential antimicrobial agent and food preservatives^17,21,70^.

The peptide sequences generated using LC–MS/MS coupled to a quadrupole time-of-flight (Q-TOF) had aligned with *P. peoriae* IBSD35 proteome using proteomics tools^21,22,24,33,38^. The sequence alignment using the Antimicrobial Peptide Database (APD3) tool indicated that peoriaerin IBSD35 is 38.23% similar to fermentin from *Lactobacillus fermentum*^20,25^. Peoriaerin IBSD35 is a trans-membrane helix with an extended strand of 10.71%, beta-turn of 21.43%, and a random coil of 64.00% (Fig. 3, Supplementary Fig. S2). It has a high percentage of helix and residues embedded in the membrane, suggesting at least some part of the sequence may embed in the hydrophobic region of the membrane^20^. Peoriaerin IBSD35 has a total net charge of + 2 and a theoretical pI of 8.86. These properties indicated an antimicrobial peptide nature. The aliphatic index was 56.07, and the grand average of the hydropathicity index (GRAVY) was − 0.132^25^. Antimicrobial action was visible after 1 h of incubation. The slower kinetics of killing suggested that peoriaerin IBSD35 might penetrate the cell membrane and transport it inside the cell cytoplasm to cause the effect^18^. However, the leakage of DNA and proteins was lacking.

The draft genome sequencing and analysis of *P. peoriae* IBSD35 provided insights into its endophytic characters, antimicrobial traits, and interaction with the host plant^10,11,13^. The characteristics contributing to strain IBSD35 epiphytic fitness are iron acquisition, capacity to colonize plants, and stress-tolerant. Additional functional genes suggested *P. peoriae* IBSD35’s close association with the environment through nitrogen fixation^14,37,40^. However, there are about 980 (18.50%) protein-coding genes without assigning any function, as per IMG AP v.4.16.6 (Table S4), and their relationship to molecular networks of the database is unknown^28,29,49^. Possibly, they may be endophytic specific, which needs further study to ascertain.

The genes encoding flagellins and flagellar motor proteins are visible in *P. peoriae* IBSD35. They helped bacterial motility in the plant-associated lifestyle^28,46,71^. The flagellin proteins (Flg) also facilitated the endophyte to pass through the first line of the plant immune defense system. Moreover, the presence of flagellin (*fli*C), glycerol kinase [EC: 2.7.1.30], elongation factor (Tu), and molecular chaperone (*htp*G) are crucial for plant-pathogen interaction indicating *P. peoriae* IBSD35 close association with the host plant^72^. Generally, motility enabled bacteria to move and colonize plants and systematically spread within the plant, promoting the endophytic lifestyle. The TCS genes reinforced traits needed for thriving in heterogeneous, highly competitive, and adverse environments^44,73^. The proteins or SMs might transport from the bacterial cytoplasm through its Tat and Sec secretion systems^61,74^.

The TCS represented a primary means by which an organism sensed and responded to stress and changing physiological environments^45,71^. The putative genes encoding small acid-soluble proteins (SASPs) annotated in *P. peoriae* IBSD35 generally helped the bacterial endospore DNA against heat, UV-radiation, and enzymic degradation^45^. They are responsible for sporulation septum, engulfment, spore morphogenesis, and germination in harsh environments. The genome ATP-binding cassette (ABC) transporter proteins are other plausible candidates for genes mediating the export of small molecules across the cells^44^. The presence of siderophores and genes conferring tolerance to oxidative stress in the strain IBSD35 genome supported the proposed importance of oxidative stress tolerance to fitness in symbiotic association. It is consistent with *P. peoriae* IBSD35 endophytic lifestyle^60,61^. However, there was no evidence of the biosynthesis of phytotoxins or cellulases.

The KEGG pathway database analysis (www.kegg.jp/kegg/kegg1.html) indicated that carbohydrate and amino acid metabolism accounted for a large proportion of the *P. peoriae* IBSD35 genome^44,45,50^, and its CCM network analysis included GG, TCA, PPP metabolites, and pyruvate metabolism^57^. Most organisms have shared a common core to their metabolic networks^58,59^. The CCM pathways convert sugars into metabolic precursors and provide the entire cell biomass, including the SMs^57^. *P. peoriae* IBSD35 harbored a collection of metabolite and drug efflux systems and bioremediation gene clusters which indicated a need to protect against toxic concentrations of metabolites or metabolic analogs^54^. It has shown the strain’s capability for bioremediation, survival defense against oxidative stress, and production of antimicrobial compounds and toxins. A repertoire of NRPS (72%) and their expressions might neutralize the competing microbes and antagonistic biomolecules. Gene resistance to β-lactam, cationic AMPs, and antifolate with the antibiotic BGCs in the strain IBSD35 genome supported that the clusters might involve in antibiotic production, which is vital to avoid self-toxicity from its own antibiotics production^54,55^.

The SMs gene clusters predicted from the *P. peoriae* IBSD35 genome showed low similarity with other BGCs. It exhibited 100% similarity for fusaricidin and polymyxin BGCs in the MIBiG cluster database (Table 4)^50,51,75^. A repertoire of NRPS SMs might help microbes in existence and communication, and their elucidation will facilitate the finding of novel bio-molecules^52^. They might also provide a competitive advantage in the battle for resources and adaptive mechanisms for survival in host plant cells or tissues^52,53,55^. The different number of BGCs in the analysis of the genomes reflected their habitats, genetics, and functional differences. Thus, a difference in genomes’ SM is a difference in environment and resource competition^55^. Generally, two closely related strains share most of their genes and will not be unique to the individual species^76,77^. The in-silico identification of PKS, NRPS, or SM gene clusters revealed considerable differences between the closely related species supporting that *P. peoriae* IBSD35 is a unique species slightly different from free-living or symbiotic rhizobium bacteria.

The terpenoids and polyketides biosynthetic are the backbones of other SMs^78^. Pathwhiz has been used to illustrate the type II polyketide biosynthesis pathway of the *P. peoriae* IBSD35 genome predicting its proteins, enzymes, SMs, and other products^79^. Dihydrokalafungin, oxytetracycline, tetracycline, nogalavinone, elloramycin, and tetranomycin F are predicted to be derived from simple carbon building blocks of the pathway (Supplementary Fig. S7). The KEGG pathway analysis (www.kegg.jp/kegg/kegg1.html) indicated that terpenoids have derived from 2-C-methyl-d-erythritol 4-phosphate/1-deoxy-d-xylulose 5-phosphate (MEP/DOXP) pathway in *P. peoriae* IBSD35. The non-mevalonate Pathway of strain IBSD35 has the potential to produce isoprenoids, antibiotics, and anticancer alkaloids^80^. However, the expressions of SM clusters are typically under environmental and developmental control mediated by complex regulatory cascades that relay signals to the pathway-specific switches^53^.

The *P. peoriae* IBSD35 genome harbored the putative genes of the nitrogen cycle. Nitrogen fixation by nitrate to ammonia and other biologically reduced forms might incorporate into amino acids and other vital compounds through nitrogen metabolism^40^. Nitrogen fixation is energetically costly, and therefore, it is tightly regulated at the transcriptional level by sophisticated regulatory networks that respond to multiple environmental cues^40,41,63^. However, the nitrogen fixation regulator (*nif*L–*nif*A) was not visible, but it was difficult to discern how oxygen levels affect the regulation of nitrogen fixation in our present study (www.kegg.jp/kegg/kegg1.html). However, it is clear that the nitrogen fixation is different from those previously described for other γ-proteobacteria and possibly depends on the host plant for its nitrogen source^63^. It is one of the unique characteristics of *P. peoriae* IBSD35 endophytic traits.

The Type (Strain) Genome Server annotation (TYGS) and PGAP annotation predicted that the strain IBSD35 has a close species relation with other *P. peoriae* strains. Phylogenetically, *P. peoriae* IBSD35 is closely related to *P. peoriae* FSL J3-0120. However, the protein Basic Local Alignment Search Tool (BLAST) indicated *P. peoriae* IBSD35 close similarity with *P. polymyxa* SC2 (E-value: 0.0, Score: 2273, Ident.: 98.9%). Therefore, the understudy genome is within the species of *P. peoriae* and related to the *P. polymyxa* species. The genomic data quality and analysis need further improvement as some of the selected genomes are draft and limited for web server analysis. The variation in genome size suggested that different strains of the same species from different habitats potentially produced their own unique NRPS products. Several gene clusters are common in selected *Paenibacillus* species suggesting their conservation in the lineage^49^. *Paenibacillus* has only been designated a separate species since 1993^12,13^. Some lineage-specific clusters and the predicted unique clusters in our understudy genome could be involved in SMs biosynthesis^12^.

The unique genes or 2125 single-copy genes from the six *Paenibacillus* genomes might encode some of the functions in the cell, as they are found only in one organism. These genes might be involved in environmental adaptation, specific competition with other species, or speciation^55,76^. The synteny dot plot indicated *P. peoriae* IBSD35 resemblance to its genus. There is also evidence of gene sharing (Mitogen-activated protein kinase (MAPK) pathway from plant, nitrogen fixation gene cluster) and horizontal transfer of genes.

Given the *P. peoriae* IBSD35 potential applications in agriculture, medicine, industries, and bio-remedy^14^, its genome sequencing and its extracellular metabolite analysis are crucial to the basic and applied research to find novel antimicrobial agents. Genome mining has enabled the prediction of the putative BGC region in the *P. peoriae* IBSD35 by linking a protein group from LC–MS/MS data and connecting these natural products to the genes^24^. The genome information and the proteomics analysis provided here will further enhance the study of environmental information processing and its antimicrobial pathways^55,64,65,81,82^. It will also facilitate the understanding of evolutionary relationships among *Paenibacillus* species. Moreover, the less explored ecological niches provide new sources of an unprecedented opportunity to find novel microbes and metabolites to counter AMR to our already exhausted sources of bio-molecules.

## Conclusions

*Paenibacillus peoriae* IBSD35 is an endophytic bacterium. It is a mesophile, endospore forming, and neutralophile bacterium. It has a linear DNA topology with a total length of 5,862,582 bp. It harbored 25 SMs BGCs, out of which 18 BGCs are NRPS. The *P. peoriae* IBSD35 closest species reference genome is *P. peoriae* KCTC 3763 with a symmetrical identity of 62.7495% and a symmetrical identity of 89.4884% with *P. peoriae* FSL J3-0120. The CFS of *P. peoriae* IBSD35 exhibited a broad spectrum of antimicrobial activity against *S. aureus* ATCC 25923, *E. coli* ATCC 25922, *C. albicans* ATCC 10231, *K. pneumoniae* ATCC 4352, and *S. typhimurium* ATCC 14028.

*P. peoriae* IBSD35 inoculum's optimum growth pH was observed at 6.8–7. It is a mesophile bacterium that grows best at 38 °C. The supernatant antimicrobial activity arbitrary unit was 1600 AU ml^−1^. The putative extracellular AMPs were purified using salting out and chromatography. A 15 proteins list and AMP sequences have predicted from LC–MS coupled to Q-TOF. An AMP from the protein lists with accession no. A0A2S6NUT6 was selected. It has been given the name peoriaerin IBSD35, based on its source name. The amino acid sequence of peoriaerin IBSD35 is AAGIQAQAGFGLSDSIQGTGKQKCSFCK with a MW of 2802.17 Da. Its sequence backbone has a large portion of helix and residues embedded in the membrane. It exhibited high antimicrobial activity against *S. aureus* ATCC 25923 at range of 0.04 µg µl^−1^.

*Paenibacillus peoriae* IBSD35 exhibited a close phylogenetic relationship with *P. peoriae *FSL J3-0120 and *P. polymyxa* CR1. The genome analysis indicated that it harbored nitrogen fixation genes and other putative nitrogen-fixing proteins but without a nitrogen fixation regulator (*nif*L/A). Its genome has a repertoire of 18 NRPS BGCs. The type II polyketide biosynthesis pathway revealed that terpenoids might be exclusively through (MEP/DOXP) pathway in *P. peoriae* IBSD35. Combining the genomics, mass spectrometry, and proteomics results obtained from *P. peoriae* IBSD35 through its extracellular biomolecules will enhance our search for AMPs that are stable, specific, and effective to counter AMR. The potential for applying comparative genomics to get evolutionary insights and discover novel AMPs from less explored regions will increase the probability of finding novel bio-molecules.

## Materials and methods

### Plant ethics statements

*Millettia pachycarpa* plant was used to isolate an endophyte, *Paenibacillus peoriae* IBSD35 which is used in this study. It is a commonly found wild species in Ukhrul, Manipur, India. Experimental research and field studies on plants including the collection of plant is complied with National guidelines and legislation. *Millettia pachycarpa* is not included in the list of IUCN Species at Risk of Extinction.

The stem of *Millettia pachycarpa* plant was collected from Phalee village (25.143524 N & 94.28334 E, 1533.14 M), Ukhrul, Manipur, India. Permissions to collect plant material were obtained from Phalee BMC by payment of fee as per NBA guidelines for collecting plant from area falls within the territorial jurisdiction under sub-section (3) of section 41 of the Act (Biological Diversity Act, 2002).

### *P. peoriae* IBSD35 culture and its morphology and genomic characters

An endophytic bacterium *P. peoriae* IBSD35 isolated from the stem of *M. pachycarpa* in our previous experiment was picked in our present context^7–9,66^. It was sub-cultured from 15% phosphate buffer saline (PBS) glycerol stock preserved in the Institute of Bioresources and Sustainable Development-Microbial Resources Division (IBSD-MRC) repository (Accession No. MRC-75001) by thawing for 5 min at room temperature (27 °C)^83^. A 100 µl of stock culture was pipetted using a micropipette and spread on a 25 ml LB agar plate. It was incubated at 38 °C to check the contamination and colony purity^84^. A single colony was streaked and sub-cultured repeatedly to maintain its growth curve character, colony purity, viability, and productivity^83,85^. The cut-well agar diffusion antimicrobial assay against *S. aureus* ATCC 25923 was performed^86^. Chemicals were added to the inoculum to avoid bacterial contamination in the fermentation system from instruments, flasks, nutrients, and pipetting.

The strain IBSD35 pure colony was smeared on a glass slide and observed under the phase-contrast microscope (Zeiss Imager. Z2)^85^. The gDNA was extracted from an overnight grown culture using the cetyl trimethyl ammonium bromide (CTAB) method^40^. The quality and quantity of the gDNA were determined by A260/A280 ratio using UV-spectrophotometer (Nanodrop, Shimadzu Biotech Bio Spec-nano) and assessed in a 0.8% agarose gel electrophoresis. The genomic and molecular characters of the genome were analyzed by draft genome sequencing using Illumina HiSeq 2500 platform^87^.

### Screening of antimicrobial activity

A 200 ml of *P. peoriae* IBSD35 inoculums was harvested by centrifugation (Sorvall ST 16R Centrifuge) at 3200×*g* for 15 min at 4 °C, and the supernatant was collected in an Eppendorf tube^88^. The spectrum of antimicrobial activity was tested using cut-well agar diffusion bioassay against the representative Gram-positive, Gram-negative, and fungal test pathogens (*S. aureus* ATCC 25923, *E. coli* ATCC 25922, *C. albicans* ATCC 10231) with a slight modification of Clinical and Laboratory Standard Institute (CSLI) methods^89–91^. The test pathogen lawn was prepared by spreading 20 µl of pathogen inoculums (0.5 Macfarland standard) over the Muller Hilton Agar (MHA) plate surface. A diameter of 3–5 mm was punched on the MHA surface with a sterile tip, and the wells were loaded with the test solution (20–100 µl) at desired concentration^86^. The MHA plates were incubated at 37 °C overnight as per the test pathogens’ best growth condition. The activity zones of inhibition diameter were measured to quantify the antimicrobial activity.

### Optimum growth pH and temperature for antimicrobial production

2 l of the production medium (pH 7 ± 2) was inoculated with an overnight grown culture of *P. peoriae* IBSD35 and incubated in a shaker incubator at 80 rpm (Eppendorf innova R42 Rotary shaker). Its optimum pH, OD, and temperature concerning antimicrobial activity were determined by aseptically aspirating out the inoculum from the fermentation broth^66^. The production medium was harvested by centrifugation at 3200×*g* for 15 min at 4 °C. The supernatant antimicrobial activity was tested by serial dilution, and its arbitrary unit was calculated against *S. aureus* ATCC 25923^31^. To rule out any false-positive inhibition caused by acid production and catalytic enzyme, 0.6% of calcium carbonate and a unit (10 mg/ml) of 0.01% catalase were added to the production medium^84^. Toluene was added to avoid contamination from other microbes^88^.

### Precipitation and purification of the antimicrobial protein mixture

The 6-day-old inoculum of 2 l of CFS was harvested from the 6-day-old inoculums by centrifugation at 3200×*g* for 15 min at 4 °C. Its pH was adjusted to 3 with 2 N hydrochloric acids (HCl) to kill the experimental strain and re-adjusted to the original harvesting state (pH 6.8) after 4 h by adding 2 N sodium hydroxide (NaOH)^88^. The supernatant pH was re-adjusted to the original harvesting state (pH 6.8) by adding 2 N NaOH. The CFS was filtered through a 0.2 µm filter (Avixa) and precipitated in 70% ammonium sulfate (NH_4_)_2_SO_4_) followed by centrifugation (3200×*g*, 35 min at 4 °C)^92^. The precipitated protein from centrifugation was collected. The resulting pellet was re-suspended in 30 ml distilled water and dialyzed using PBS in a dialysis membrane (12.4 kDa cut-off) with intermittent buffer changing^36^.

The pellet was re-dissolved in 30 ml of distilled water and loaded in diethylaminoethyl cellulose (DEAE-C (Sigma Aldrich). A weakly positive resin, DEAE-cellulose was loaded for anion exchange column chromatography^93^. Sample was eluted out using 0.1, 0.2, 0.3, 0.4, 0.5 and 0.7 M or 100–700 mM NaCl gradient elution and the OD were recorded at selected wavelength of λ205, λ214, and λ280^15,16^. The fractions were pooled together and lyophilized into powder form and tested for retention of antimicrobial activity according to the CLSI assay^89,90^. Fractions showing antimicrobial activity were pooled together for lyophilization and further purified with RP-HPLC^94^.

### RP-HPLC purification of protein mixture

The partially purified sample was again purified using ZORBAX 300 SB-C-18 semi-preparative (9.4 × 250 mm) 5 microns^91,94^. The column was irrigated with 0.1% TFA in 20% ACN for 45 min at an isocratic flow rate. The sample (150 µl per experiment) was loaded on the HPLC (UFLC Shimazdu) with semi-preparative Agilent ZORBAX 300SB reverse-phase C-18 of 5 µm and 9.4 × 250 mm column and run with 0.1% ion-pairing reagent, trifluoroacetic acid (TFA)^68,94^. The RP-HPLC mobile phase consisted of (i) HPLC-grade water containing 0.1% TFA and (ii) 20% acetonitrile (ACN) in an isocratic system at a flow rate of 2.5 ml min^−1^^93^. Eluents were monitored at a wavelength of λ205, λ214, and λ280 using a UV-spectrometer (Eppendorf, Biospectrometer) and collected manually. The fractions which exhibited higher antimicrobial activity were pooled together and collected separately for further analysis. The total protein concentration and activity, specific activity, yield percentage, and purification fold of the extract compared to the initial starting crude extract were calculated using UV-Spectroscopy^15,16^. The UV-spectrophotometric method for antimicrobial protein concentration was calculated as: concentration (mg/ml) = (1.55 × A280) (0.76 × A260) × dilution factor^69^.

### The effect of external factors on sample antimicrobial activity

The sample was evaluated for sensitivity to degradative enzymes, detergents, organic solvents, temperature, and pH changes^78,84^. The retention of antimicrobial activity after the treatment was qualitatively determined through cut-well agar diffusion bioassay against the reference pathogen, *S. aureus* ATCC 25923 as described earlier^91^. For the pH stability test, the sample dissolved in dist. water was pre-adjusted to pH 2.0–8.9, followed by incubation at 38 °C for 12 h. The sample temperature sensitivity was tested by exposing to temperatures ranging from 20 to 100 °C at the interval of 10 °C for 2–3 h, and additionally at 121 °C for 15 min by autoclaving^86,91^.

The samples were treated with different degradative enzymes at the concentration of 1 mg ml^−1^ for 2–3 h at room temperature to test the stability against enzymes^84^. The organic solvent and test sample were mixed in a 1:1 ratio and incubated at 27 °C to evaporate all the organic solvent, and the samples were assessed for retention of antimicrobial activity. The samples were treated with detergents in a 1:1 ratio and checked for retention of antimicrobial activity^86,90^. Negative control consists of PBS (pH 7) and the untreated sample solution was used as a positive control.

### Mass spectrometric analysis of peptide mixture

The experiment was performed using EASY-nLC 1000 system (Thermo Fisher Scientific) coupled to a Q-Exactive mass spectrometer (Thermo Fisher Scientific) equipped with a nano-electrospray ion source^23,24^. 1.0 µg of the RP-HPLC purified peptide mixture was resolved using a 15 cm PicoFrit column (360 µm outer diameter, 75 µm inner diameter, 10 µm tip) filled with 1.9 µm of C18-resin (Dr. Maeisch, Germany). The peptides were loaded with buffer A and eluted with a 0–40% gradient of buffer B (95% ACN, 0.1% formic acid) at a flow rate of 300 nl/min for 90 min^24^. The source heater was set at 300 °C to turn the sample into a vapor for analysis. The UPLC-separated samples were then analyzed by electrospray ionization at 4.5 kV and the peptides were dissociated with collision induced dissociation (CID) for MS/MS spectra^95^. MS data was acquired using a data-dependent top10 method dynamically choosing the most abundant precursor ions from the survey scan.

One sample was processed and 1 RAW file generated was analyzed with a Proteome Discoverer against *P. peoriae* IBSD35 Uniprot reference proteome database^33^. For the Sequest search, the precursor and fragment mass tolerances were set at 10 ppm and 0.5 Da, respectively. Trypsin was used to generate peptides, carbamidomethyl on cysteine as fixed modification, and oxidation of methionine, and N-terminal acetylation was considered as variable modifications for database search. Both peptide spectrum match (PSM) and protein false discovery rate (FDR) were set to 0.01 FDR.

Physiochemical properties of the protein and AMPs were analyzed using the ExPASy-ProtParam tool (http://web.expasy.org/protparam) and APD3 Antimicrobial Peptide Calculator^23,25^. The secondary structure was analyzed using SOPMA^96^. The 3-D conformation structure of the peptide was predicted using PEP-FOLD 3 de novo modeling and deposited in ModelArchive^26,27^. The model was built based on the target-template alignment using ProMod3^97^.

### Peoraerin IBSD35 mode of action imaging through scanning electron microscopy

The AMP mode of action against the test pathogens was checked by the absorbance at λ260 and λ280 with UV-spectrometry. A direct visualization of peptide-induced *S. aureus* cellular damage and morphological changes were evaluated by SEM^98^. The samples were prepared by incubating *S. aureus* ATTC 25923 with the test sample as described earlier^89,90^. *S. aureus* were treated with antimicrobial agent at 1× MIC for 2–3 h and incubated at 30 °C ± 0.2. The inoculants were harvested by centrifugation as described earlier^87^. The pellet was washed with 0.1 M PBS (pH 7.2) and fixed in 2.5% glutaraldehyde and 2% paraformaldehyde made in PBS^97^. The fixed sample was further processed for coating and imaging^99^. The test pathogen without any treatment served as the experimental control.

### Genome sequencing and assembly

The gDNA integrity was confirmed using a 0.8% agarose gel electrophoresis^97^. The draft genome sequencing was performed through Illumina HiSeq 2500 system^34^. The cleaned reads were subjected to the Kmer genie to predict the optimal k value and assembly size^100^. De novo assembly was performed using ABySS v.2.0^42^. The predicted genes were matched with bacterial proteins from the Uniprot database using the BLASTX program^101^. Genes that could establish homology relationships (E-value ≤ 10^−5^ and similarity score ≥ 40%) were retained in the annotation pipeline for further annotation while the others remain unannotated^28–30^.

### Genome annotation and bioinformatics analysis

JGI-IMG Annotation Pipeline v.4.16.6^28,29^ and the NCBI PGAP were used for gene annotation^30^. The gene functional prediction was performed by BLAST against the databases^101^.

### Comparative genomic analysis

The close strains of *P. peoriae* IBSD35 (NZ_PTJM01000000)^7,66^ were selected from the NCBI database (*P. peoriae* KCTC 3763^102^, *P. polymyxa* E681(NZ_CP048793)^75^, *P. polymyxa* SC2 (NC_014622)^103,104^, *P. vortex* V453 (NZ_ADHJ01000000)^105^, *P. terrae* HPL-003(NC_016641)^106^, *Paenibacillus* sp. JDR-2 (NC_012914)^107^, *P. polymyxa* CR1(NC_023037)^108^, *B. subtilis subtilis* AG1839 (NZ_CP008698)^109^, *S. coelicolor* A3(2) (AL645882)^110^, and *P. peoriae* HS311 (NZ_CP011512)). *Bacillus subtilis subtilis* AG1839 is selected because *Paenibacillus* represents a new genus from *Bacillus*^12,13^. *S. coelicolor* A3(2) is the model organism for SM BGC studies. *P. peoriae* HS311 represents the completely sequenced genomes of *Paenibacillus* and serves as the reference for the natural variation within the genus. *P. peoriae* KCTC 3763 represents the closest species reference genome with a symmetrical identity of 62.7495%.

OrthoVenn was used for Venn diagram drawing which is based on the protein sequences clusters (E-value = 1e−2 and inflation value = 1.5)^76^. Web Gene Ontology Annotation Plot (WEGO) was used to compare the provided gene datasets and plot the distribution of GO annotation into a histogram^38^. SMs biosynthesis gene clusters were analyzed with AntiSMASH V.5 and JGI-IMG/ABC, and the type II polyketide biosynthesis pathway was drawn using KEGG and PathWhiz tool^43,50,51,79^. All comparative analyses were performed using IMG/M and RAST tools^29,37,39^.

### Genome-based taxonomy and phylogenetic tree

Genome-based taxonomy was determined using TYGS which infers genome-scale phylogenies and estimates for species and subspecies boundaries and automatically determined closest type genome sequences^111,112^. The pair-wise digital DNA:DNA hybridization (dDDH) values between user genomes and the selected types-strain genomes were performed^77^. The dDDH values are provided along with their confidence intervals (C.I.) and difference of G + C% using the Genome BLAST Distance Phylogeny (GBDP) formula d5^77,78^. The average nucleotide identity with the closely related species was determined using the OAT tool^113^. The phylogenetic tree was inferred with FastME 2.1.6.1 from GBDP distances calculated from genome sequences^114^. The AMP model was built based on the target-template alignment using ProMod3^97^.

## Supplementary Information

Supplementary Information.

## Data Availability

The datasets generated or analyzed during the current study are given in the table form with the name, accession number, and the database or web link.

**Table:** 

Name	Accession number	Database/web link
*Paenibacillus peoriae* IBSD35	BioProject: PRJNA224116	NCBI
Unknown protein from LC–MS experiment	A0A2S6NUT6	Uniprot
Peoriaerin IBSD35	ma-id7l4 (DOI 10.5452ma-id7l4)	ModelArchive
Nitrogen metabolism (map00910)	Ref: 210413	https://www.kegg.jp/kegg/
Biosynthesis of type II polyketide backbone	PW122264	https://smpdb.ca/pathwhiz/pathways/PW122264

ma-id7l4—(DOI 10.5452ma-id7l4) will be activated when we submitted the accepted paper to ModelArchive.

Ref: 210413—A Permission document from KEGG is provided separately. ma-id7l4—(DOI 10.5452ma-id7l4) will be activated when we submitted the accepted paper to ModelArchive. Ref: 210413—A Permission document from KEGG is provided separately.
