# Supplementary material for: Integrated genomics and proteomics analysis of Paenibacillus peoriae IBSD35 and insights into its antimicrobial characteristics
Source: Sci Rep. 2022 Nov 7;12:18861. doi: 10.1038/s41598-022-23613-y (PMC9640621; doi:10.1038/s41598-022-23613-y)
Supplement: Supplementary file 1 — Supplementary Information. [file 41598_2022_23613_MOESM1_ESM.docx]

**Supplementary File**

**Supplementary Table**

**Table S1.** **Purification of antimicrobial biomolecules from crude extract, and the chromatography eluents optical densities at λ_205,_ λ_214_, and λ_208_ with their respective antimicrobial activity.** (NA- non applicable, O-zero values, (-) - negative antimicrobial activity, and (+) - positive antimicrobial activity).

| **Sample** | **Code** | **Amount**  **(ml)** | **OD λ_205_** | **OD λ_214_** | **OD**  **λ_280_** | **Antimicrobial activity** |
| --- | --- | --- | --- | --- | --- | --- |
| Crude | C | 10 | NA | NA |  | + |
| 70% Ammonium sulfate precipitation | A | 30 | NA | NA |  | + |
| 200mM NaCl | F2 | 8 | 0 | 0 | 0 | - |
| 400mM NaCl | F3 | 8 | 0 | 0 | 2.410 | - |
| 700mM NaCl | F4 | 20 | 0 | 1.417 | 2.803 | + |
| HPLC peak 4 | P4 | 1.5 | 0.320 | 0.136 | 0.083 | + |
| HPLC peak 5 | P5 | 1.5 | 0.423 | 0.285 | 0.101 | + |

**Table S2. RP-HPLC profile of the partially purified sample.**

| **Peak** | **Ret time** | **Area** | **Height** | **Area %** | **Height %** | **Peak purity index** |
| --- | --- | --- | --- | --- | --- | --- |
| P1 | 1.591 | 23994 | 855 | 0.181 | 0.051 | 0.96835 |
| P2 | 2.235 | 86257 | 8176 | 0.650 | 0.492 | 0.70032 |
| P3 | 2.573 | 32648 | 3305 | 0.246 | 0.199 | -0.38205 |
| P4 | 4.165 | 5007534 | 779925 | 37.722 | 46.901 | 0.86310 |
| P5 | 4.567 | 1084718 | 62409 | 8.171 | 3.753 | 0.95597 |
| P6 | 4.935 | 4571141 | 746722 | 34.434 | 44.904 | 0.93576 |
| P7 | 5.408 | 4967 | 1193 | 0.037 | 0.072 | -0.22884 |
| P8 | 5.654 | 47211 | 7080 | 0.356 | 0.426 | 0.96136 |
| P9 | 6.050 | 104877 | 10700 | 0.790 | 0.643 | 0.23456S |
| P10 | 6.226 | 12476 | 2401 | 0.094 | 0.144 | 0.25509 |

**Table S3. Antimicrobial activity after treatment with degrading enzymes, inorganic solvent, detergents, pH, and temperature**. The zone of inhibition which is more than 6 mm in diameter is considered a positive retention of antimicrobial activity. PBS is used a control. Ampicillin is used as a positive control.

| **Treatment** | **Zone of inhibition diameter (mm)** |
| --- | --- |
| **Enzyme** (1mg/ml) |  |
| Proteinase K | 11 |
| Catalase | 10 |
| Pectinase | 9 |
| Cellulase | 9 |
| Pepsin | 10 |
| **Detergents (1:1)** |  |
| SDS | 14 |
| Triton X | 14 |
| Tween 20 | 13 |
| Tween 80 | 12 |
| **Inorganic solvents (1:1)** |  |
| Methanol | 9 |
| Butanol | 10 |
| Ethyl acetate | 12 |
| Petroleum ether | 8 |
| Isopropanol | 8 |
| Acetone | 12 |
| **Temperature (1:1)** |  |
| 20 | 11 |
| 40 | 10 |
| 60 | 10 |
| 80 | 11 |
| 100 | 10 |
| 121 | 14 |
| **pH (1:1)** |  |
| 2 | 12 |
| 5.5 | 12 |
| 6 | 13 |
| 6.4 | 12 |
| 7 | 12 |
| 7.5 | 13 |
| 8 | 9 |
| 8.9 | 9 |
| **PBS*** | 0 |
| Untreated sample solution* | 0 |
| **Amp*** (1mg/ml) | 20 |

**Table S4. General features of *P. peoriae* IBSD35 genome annotated by NCBI Prokaryotic Genome Annotation Pipeline.**

| **Category** | **Number** |
| --- | --- |
| Genome size (nt) | 5,862,582 |
| G + C content (%) | 45.6 |
| Chromosome topology | Linear |
| Protein coding gene | 4983 |
| Genes with assigned function | 5245 |
| Scaffolds | 60 |
| Contigs | 65 |
| Contig N50 | 400,732 |
| Contig L50 | 6 |
| tRNA | 81 |
| rRNA | 9, 2, 3 (5S, 16S, 23S) |
| ncRNAs | 4 |
| Pseudogenes | 263 |
| CRISPR Arrays | 3 |

**Table S5. Nitrogen metabolism (map00910): reduction and fixation from the KEGG pathway analysis**. The kegg ontology, gene, description, and their known enzyme numbers were derived from KEGG pathway database.

| **KEGG** | **Gene** | **Description with known enzyme numbers** |
| --- | --- | --- |
| K02575 | *nrt* | MFS transporter |
| K03320 |  | NNP family, nitrate/nitrite transporter |
| K00370 | *amt* | Ammonium transporter which involved in the uptake of nitrate and nitrite |
|  | *nif*N, *nif*X, *nif*B, *nif*Q, *nif*V, *nif*Y, *nif*H | Contribute to the synthesis and insertion of FeMo-Co into nitrogenase |
| - | *nif*U, *nif*S, *nif*Z | Play an important role in synthesis of metalloclusters and that |
|  | *nif*M | Required for proper folder of nitrogenase Fe protein |
| K00370 | *nar*G, *nar*H | Nitrate reductase/nitrite oxidoreductase, alpha subunit |
| K00372 | *nas*A | Assimilatory nitrate reductase catalytic subunit [EC:1.7.99.-] |
| K00366 | *nir*A | Ferredoxin-nitrite reductase |
| K00362 | *nir*B | Nitrite reductase (NADH) large subunit |
| K05601 | *hcp* | Hydroxylamine reductase [EC:1.7.99.1] |
| K00262 | *gdh*A | Glutamate dehydrogenase (NADP+) [EC:1.4.1.4] |
| GO: [0004089](http://amigo.geneontology.org/amigo/term/GO:0004089) |  | Carbonic anhydrase [EC:4.2.1.1] |
| GO: [0004355](http://amigo.geneontology.org/amigo/term/GO:0004355), [0016040](http://amigo.geneontology.org/amigo/term/GO:0016040) |  | Glutamate synthase (NADPH/NADH) [EC:1.4.1.13 1.4.1.14] |
| K00284 |  | Glutamate synthase (ferredoxin) [EC:1.4.7.1] |
| K00373 | *Nar*J/*Nar*W | Nitrate reductase molybdenum cofactor assembly chaperone |
| K02591 | *nif*K, *nif*D | Nitrogenase (molybdenum-iron) beta chain (EC 1.18.6.1) |
| K02596 | *nif*X | Nitrogen-fixing protein |
| K04488 | *isc*U, *nif*Z | Putative nitrogen-fixing protein |
| K02596 | *nif*X | Nitrogen-fixing protein |
| K0259 | *nif*N | Nitrogenase FeMo cofactor carrier protein |
| K02587 | *nif*E | Nitrogenase FeMo cofactor scaffold and assembly protein |
| K02588 | *nif*E | Nitrogenase molybdenum-cofactor synthesis protein |
| K02585 | *nif*H | Nitrogenase iron and maturation protein |
| K02594 | *nif*B | Nitrogenase FeMo cofactor synthesis FeS core scaffold and assembly protein |
| K02594 | *nif*V | Homocitrate synthase |

**Table S6. Antimicrobial susceptibility test of RP-HPLC purified sample against different pathogens.** Its activity against different pathogens is different. Ampicillin was used as a reference. (- indicates non activity).

| **Sl. No.** | **Organisms** | **ATCC Code** | **Sample** | **Concentration**  **(µg/µl)** | **Activity (mm)** |
| --- | --- | --- | --- | --- | --- |
| 1 | *Klebsiella pneumoniae* | ATCC 4352 | F4 | 1.5 | - |
| 2 | *Pseudomonas aeruginosa* | ATCC 15442 | F4 | 1.5 | 2 |
| 3 | *Pseudomonas fluorescens* | ATCC 13525 | F4 | 1.5 | - |
| 4 | *Cryptococcus neoformans* | ATCC 14116 | F4 | 1.5 | - |
| 5 | *Shigella flexneri* | ATCC 12022 | F4 | 1.5 | 10 |
| 6 | *Candida tropicalis* | ATCC 750 | F4 | 1.5 | 15 |
| 7 | *Staphylococcus aureus* | ATCC 25923 | F4 | 1.5 | 10 |
| 8 | *Bacillus subtilis* | ATCC 6633 | F4 | 1.5 | 5 |
| 9 | *Escherichia coli* | ATCC 25922 | F4 | 1.5 | 13 |
| 10 | *Enterococcus faecalis* | ATCC 29212 | F4 | 1.5 | - |
| 11 | *Candida albicans* | ATCC 10231 | F4 | 1.5 | - |
| 12 | *Salmonella typhimurium* | ATCC 14028 | F4 | 1.5 | - |
| 13 | Antibiotics | Ampicillin | Amp | 1mg/ml | 20.3 |

**Table S7. Representative test pathogens at their optimal growth condition, media and their susceptibility to the extract from *P. peoriae* IBSD35.** Each pathogen grows differently in different media. The antimicrobial peptide acted differently toward different pathogens. The activity was measured from the zone of inhibition diameter. (*NA- Nutrient Agar, TSA- Tryptic soy agar, YM- Yeast malt extract, BHI- Brain heart infusion, LB- Luria bertani*).

| **Sl. No.** | **Organisms** | **ATTC Code** | **Media** | **Growth condition (^0^C)** | **Antimicrobial activity Diameter (mm)** |
| --- | --- | --- | --- | --- | --- |
| 1 | *Klebsiella pneumoniae* | ATCC 4352 | NA | 37 | 10 |
| 2 | *Pseudomonas aeruginosa* | ATCC 15442 | TSA | 37 | 11 |
| 3 | *Pseudomonas fluorescens* | ATCC 13525 | NA | 26 | 11 |
| 4 | *Cryptococcus neoformans* | ATCC 14116 | YM | 26 | 9 |
| 5 | *Shigella flexneri* | ATCC 12022 | NA | 37 | 8 |
| 6 | *Candida tropicalis* | ATCC 750 | YM | 25 | 10 |
| 7 | *Staphylococcus aureus* | ATCC 25923 | TSA, LB, BHI | 37 | 14 |
| 8 | *Bacillus subtilis* | ATCC 6633 | BHI | 30 | 12 |
| 9 | *Escherichia coli* | ATCC 25922 | TSA, LB, BHI | 37 | 12 |
| 10 | *Enterococcus faecalis* | ATCC 29212 | TSA | 37 | 9 |
| 11 | *Candida albicans* | ATCC 10231 | YM | 25 | 9 |
| 12 | *Salmonella typhimurium* | ATCC 14028 | NA | 37 | 13 |

**Supplementary Figure**


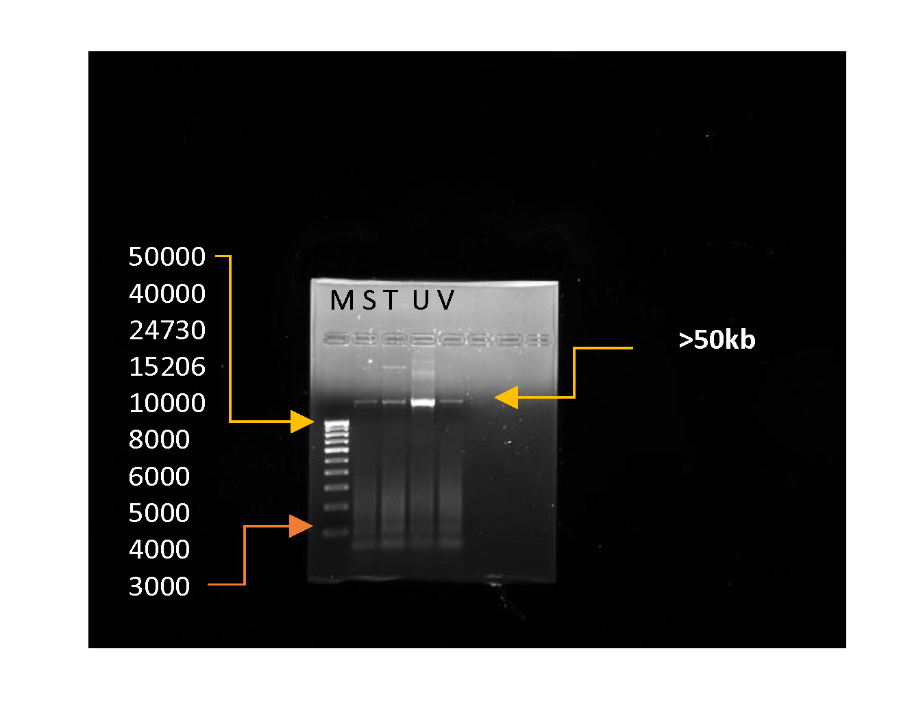


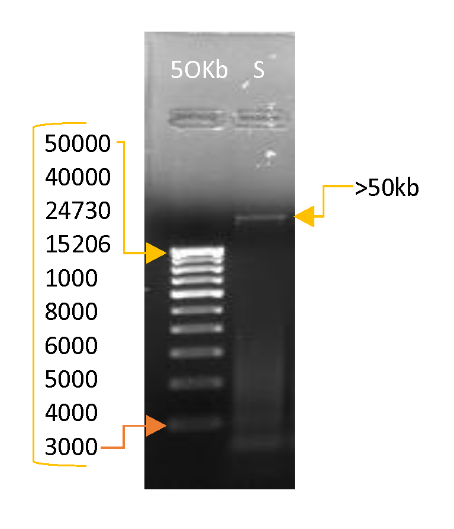


**Figure S1. The original genomic DNA gel in 0.8% agarose gel.** M is the marker lane, S is the under-study sample lane, lane V is the duplicate of S and T and U are the reference sample and the remaining lanes are blank. The genomic size is more than 50 Kbp. The original gel, labelled gel, and the edited gel are shown.


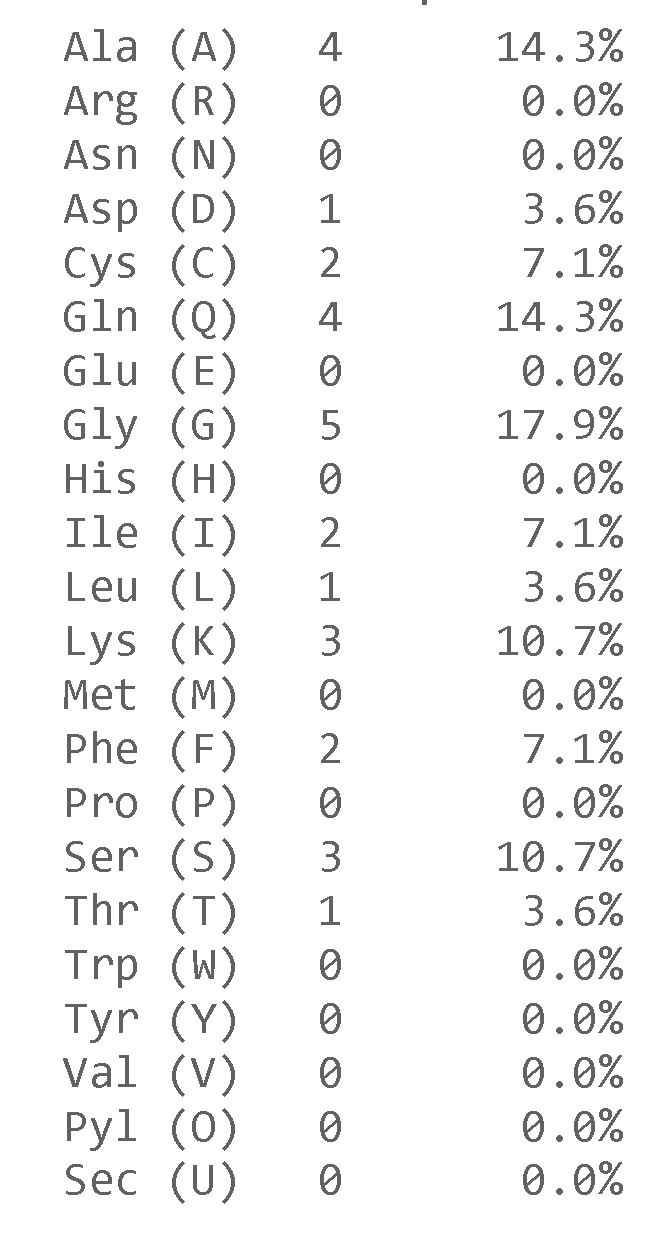


**Figure S2. The amino acid composition of peptide indicated in percentage**. Total number of negatively charged residues is 4 (Asp + Glu) and positively charged residues is 2 (Arg + Lys). The most abundant amino acids are Thr (19.5 %), Ser (17.1 %), Glu, His and Leu (7.3 %), and Cys (7.1%).

**Figure S3. Functional traits of *Paenibacillus peoriae* IBSD35 generated using BlastKOALA.** The maximum number of protein coding genes is related to genetic information process (47%), environment information processing (45%), carbohydrate metabolism protein family (42%), and signaling process (31%).


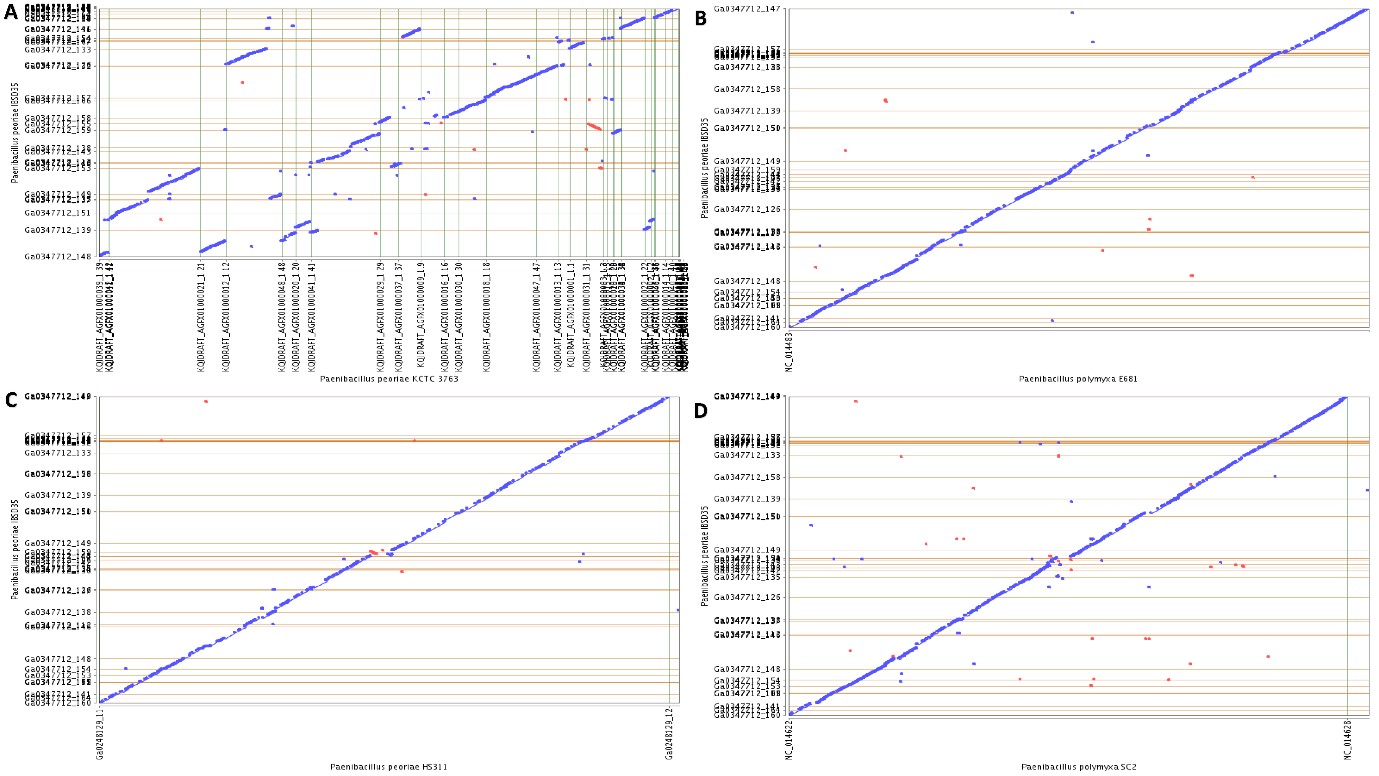


**Figure S4. Synteny dot plots of *P. peoriae* IBSD35 with its close relatives**. The X-axis and Y-axis represent the genome sequence in scaffolds and each dot represents a putative homologous match between the two comparing sequences. The dot plot consists of blue points for regions of similarity found on parallel strands (fplot) and red points for regions of similarity found on antiparallel strands (rplot). The analysis was carried out using the Dot plot from Integrated Microbial Genome (IMG) website.


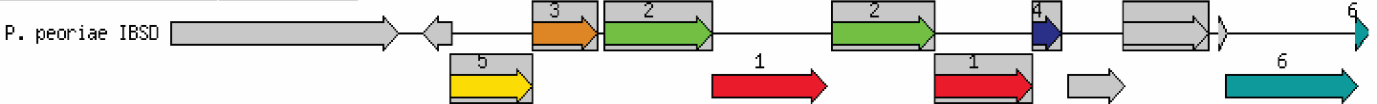


**Figure S5. Schematic diagram of (1) K02591, *nif K*; nitrogenase (molybdenum-iron) beta chain (EC 1.18.6.1) in *Paenibacillus peoriae* IBSD35 from KEGG pathway analysis. (2) Nitrogenase (molybdenum-iron) alpha chain (EC 1.18.6.1) (3) Nitrogenase (molybdenum-iron) reductase and maturation protein NifH (4) Nitrogenase FeMo-cofactor carrier protein NifX (5) Nitrogenase FeMo-cofactor synthesis FeS core scaffold and assembly protein NifB (6) Lipid A export ATP-binding/permease protein MsbA.**


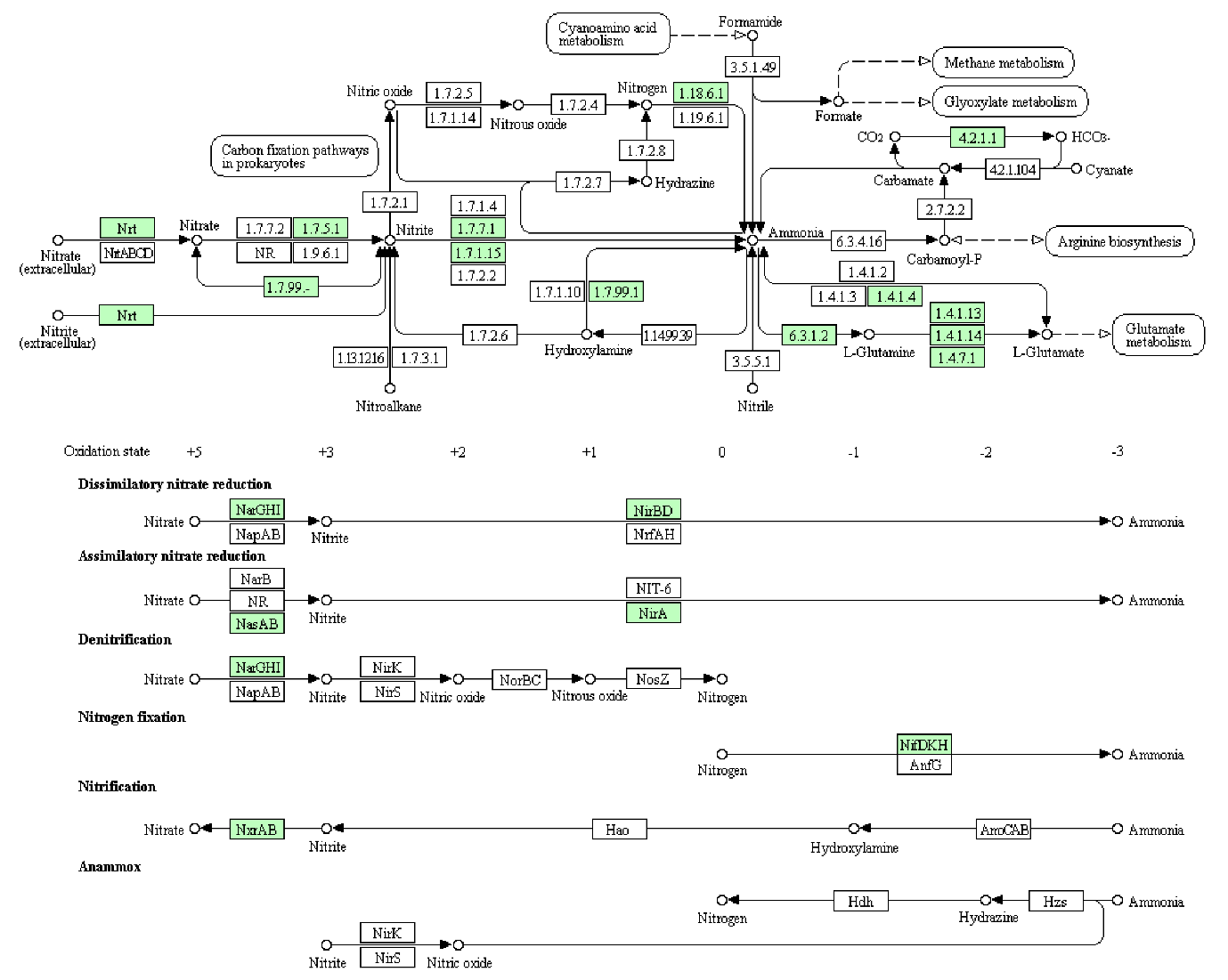


**Figure S6. Nitrogen metabolism (map00910) pathway of *Paenibacillus peoriae* IBSD35.** The light green color indicates the enzymes involves in nitrogen metabolism pathway from KEGG pathway database analysis.


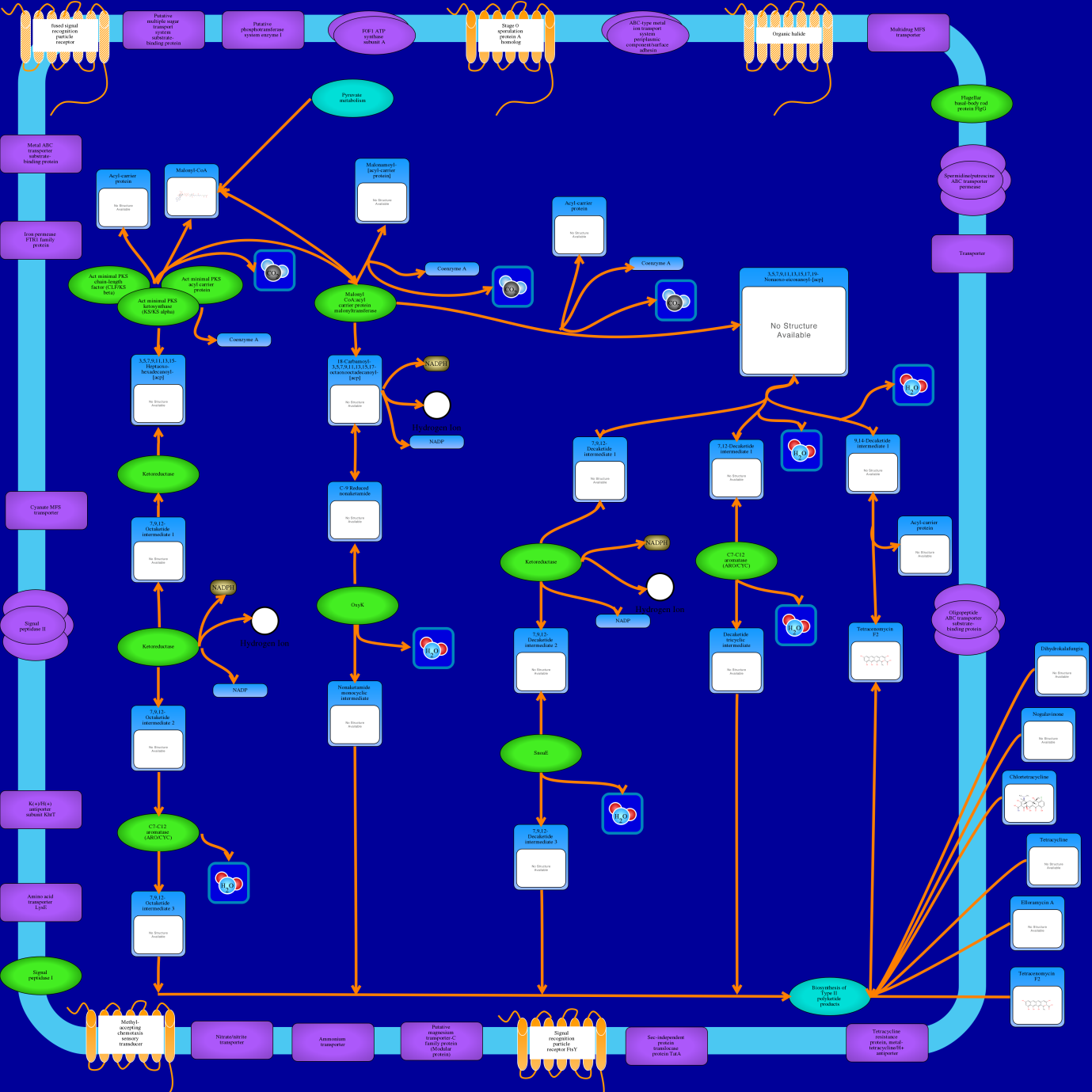


**Figure S7**. **Schematic view of secondary metabolites backbone type II polyketide biosynthesis pathway from KEGG pathway database.** The putative products are outside of the cell membrane (Red colour). The pathway was drawn using pathwhiz tool.
